# Characterization of the fecal microbiota of sows and their offspring from German commercial pig farms

**DOI:** 10.1371/journal.pone.0256112

**Published:** 2021-08-16

**Authors:** Anja Lührmann, Ksenia Ovadenko, Justinus Hellmich, Christoph Sudendey, Vitaly Belik, Jürgen Zentek, Wilfried Vahjen

**Affiliations:** 1 Department of Veterinary Medicine, Institute of Animal Nutrition, Freie Universität Berlin, Berlin, Germany; 2 Department of Veterinary Medicine, System Modeling Group, Institute of Veterinary Epidemiology and Biostatistics, Freie Universität Berlin, Berlin, Germany; University of Maine, UNITED STATES

## Abstract

Strategies to combat microbiota-associated health problems are of high interest in pig production. Successful intervention strategies with beneficial long-term effects are still missing. Most studies on pig microbiota have been conducted under standardized experimental conditions, but the situation in commercial farms differs dramatically. This study describes the fecal microbiota in German commercial pig farms under practical conditions. The study is part of the larger project “Optibiom” that aims to use bacterial composition and farm metadata to formulate tailor-made solutions for farm-specific health maintenance strategies. Special consideration is given to the sow-piglet relationship. Fecal samples from sows and their piglets were collected at two time points each in 20 different farms (sows ante- and postpartum and piglets before and after weaning). The extracted DNA was sequenced with Illumina 16S rDNA sequencing. For data analysis and visualization, differential abundance analyses, as well as hierarchical clustering and nonmetric multidimensional scaling (NMDS) were performed. A new “family unit” was implemented to compare farms based on the association between the microbiota in sows and their offspring. There are distinct changes in the microbial communities in sows before and after birth as well as in suckling and post-weaning piglets. The suckling pig microbiota is particularly different from all other groups and shows a lower bacterial diversity. While dominant genera in antepartum sows further displace the abundance of non-dominant genera postpartum, the opposite was true for piglets, where non-dominant bacteria in the suckling phase became dominant after weaning. The family unit for sows and their piglets led to separate cluster formation for some farms. The results indicate that the sow-piglet relationship is one driving force for the observed differences of the pig farms. The next step in the analysis will be the combination of metadata (feeding, housing and management practices) to find farm-specific differences that can be exploited to formulate a farm-specific health maintenance strategy.

## Introduction

Research into the intestinal microbiota of sows and their offspring has intensified during the last years, as a link between animal health and their microbiota has been established [1]. No strategy has yet been identified to successfully use microbiota modifications to guard pigs against typical problems in commercial pig production, such as high mortality of suckling piglets or post-weaning diarrhea. A new health strategy would not be limited to disease prevention, but also involve economic limits of farms, environmental concerns, and animal welfare. The first step to formulate such a strategy for future pig farming is obviously the description of the microbiota under practical farm conditions.

In recent years, microbial transmission between sow and offspring has been recognized as an important factor in the development of the microbiota in piglets [2]. One of the primary factors that govern this unique mother-child ecosystem in pigs is the close contact of the piglet with maternal microbiota during birth, and the suckling phase [3].

In piglets, the most critical phases for disease susceptibility are the suckling period and the weaning period. Already shortly after birth, neonatal diarrhea can occur, whereby the intestinal microbiota seems to play a key role [4, 5]. In the suckling period, a dysbiosis and overgrowth of bacteria like pathogenic *Escherichia coli* and *Clostridium difficile* are a major cause of severe diarrhea and piglet losses [6, 7]. Weaning stress, the introduction of solid feed and new husbandry conditions are accompanied by a shift in the intestinal microbiota composition. This can lead to strong post-weaning diarrhea [8, 9], which is most probably due to microbiota-induced susceptibilities [10]. Since early microbial colonization influences later health and performance [11], the link between sow and piglet microbiota is not to be disregarded. In this study, we, therefore, introduce a novel computational analysis of “family unit” that defines the mother-child relationship based on their fecal microbiome. To describe the fecal microbiota in sows and their offspring, 16S rDNA sequencing was performed with the intent to learn more about the mother-child relationship. Due to its comparably low costs, 16S rDNA sequencing today can be considered as a practical application for future routine diagnostics of commercial pig farms. Although a more in-depth analysis of the fecal microbiota would have been advantageous, the high sample number in this study prohibited a true metagenomic approach. Combined with a bioinformatic analysis via amplicon sequence variants (ASV) [12] and correlation to available metadata from individual farms (feeding, housing, environment, genetics), 16S rDNA sequencing may become a central parameter to describe and potentially resolve health problems in commercial pig farms. Challenges to this concept are not only the multiple factors affecting the microbiota but also the interpretation of the large amount of data. Explorative data analysis is a first step to discover possible similarities within the investigated parameters. In this regard, nonmetric multidimensional scaling (NMDS) has proven to be a more robust ordination method for microbiome data compared to other popular ordination techniques such as principal component analysis and principal coordinate analysis [13]. The standard for detection of taxonomic shifts across different samples is still the comparison of relative abundance data. However, there are high false discovery rates that lead to unreliable estimation of the real ecosystem composition [14, 15], because an increase/ decrease of the relative abundance of a given bacterial genus is, not quantitative and noise impaired. The recently presented method of differential abundance analysis circumvents associated problems of simple before/after comparison of relative abundance by introducing a reference frame that is used to express quantitative fold changes in microbiota composition [16]. Therefore, the differential abundance analysis is better suited to identify true changes in microbiota composition.

Besides difficult interpretation of sequencing results, studies of microbial shifts in sows [17, 18] and piglets [19, 20] are mostly done under standardized experimental conditions, which do not fully reflect conditions under commercial practice and their outcome on the pig microbiota [21]. As there is a lack of studies investigating the microbiome of different pig farms, effects of interventions on the microbiota cannot be determined. Therefore, in an effort to formulate tailor-made health maintenance strategies, the project “Optibiom” aims to combine farm metadata and microbial composition to elucidate possible interventions to increase health in specific farms. Within that project, the present study aimed to describe the existing situation in pig farms in Germany as a necessary first step. Data of this study will provide a basis for further investigations regarding the impact of individual farm practices.

## Methods

### Ethics statement

The present study is not an animal experiment as defined by the German Animal Welfare Act (TierSchG 2006). Fecal samples were collected non-invasively.

### Farm selection

This study was performed by selecting 20 pig farms in Germany that volunteered to cooperate in the project Optibiom for a longer sampling period and that had a steady farm structure (either a closed system or fixed supplier relationships). Furthermore, enough sows had to be available for the production cycle to guarantee a sufficient number of samples for different phases of animal production. Different situations of general intestinal health of the animals as well as performance evaluation were considered to approximate a typical field situation in Germany. The management or husbandry conditions of the farms were not changed or adapted because of the study.

### Study design and sampling

The study design was based on sampling the same sows and their offspring over a period of seven weeks during the production cycle (sows 15 ± 3d antepartum and 11 ± 3 d postpartum, piglets at the age of 11±3 d and 34±3 d). A total of 802 fresh fecal samples were acquired from 10 ± 1 sows and 10 ± 1 piglets from 20 different piglet production and rearing farms. The selection of one piglet per sow took place immediately after birth of the whole litter. The person in charge of the sows was instructed to select the strongest piglet (body condition and group rank for teats) for the study. This was to avoid a possible loss or necessary supplementary feeding due to weakness in the further course of the study. The sex was not considered for selection.

Fresh fecal samples were taken at spontaneous defecation, shock frozen in liquid nitrogen, and stored at -20°C until further processing. Due to biosecurity considerations (time interval between appointments in different farms) and matching of sampling appointments to the production rhythms of the different farms, the entire sampling period lasted nine months.

### DNA extraction and 16S rDNA sequencing

Total DNA was extracted from 0.25 g feces with a commercial extraction kit (QIAamp PowerFecal Pro DNA Kit, Qiagen, Hilden, Germany) in accordance with the manufacturer’s instructions with an additional lysis step at 65°C. Furthermore, due to the high fat content in feces of suckling piglets, the amount of the lysis buffer was increased by 200 μl in the first step. For the homogenization step the FastPrep-24^TM^ 5G (M.P. Biomedicals LLC, Santa Ana, California, USA) was used at speed of 6 m/s for 10 min (4 times 5 x 30 s and 15 s pause time). DNA content was determined via fluorometry and extracts were stored at –30°C until further analysis. DNA extracts were subjected to amplicon sequencing using an Illumina NextSeq500 sequencer (LGC, Berlin, Germany) with 150 bp-paired reads using 16S rDNA primers 341f and 785r. Demultiplexing was achieved with Illumina bcl2fastq (v. 2.17.1.14); combination of paired reads was done with BBMerge (v. 34.48).

### Bioinformatic analysis

The resulting 16S-rDNA sequences were analyzed using the QIIME2 pipeline [22] and the SILVA SSU database [23]. Quality control and determination of sequence counts were performed using the DADA2 [24]. Further details were previously described [25]. The bacterial diversity measures Richness, Shannon index, and Evenness were calculated from ASV level data.

### Relative abundance analyses

Results for relative abundance are comprehensively presented as means and standard deviations in S1–S6 Tables. Due to the non-normal distribution of the data, the Mann–Whitney test was chosen to compare the relative abundances for sow and piglet data. Statistical procedures were performed using the IBM SPSS Statistics software Version 25 (IBM, Chicago, USA). A level of 95% confidence was deemed as significantly different.

**Fig 1:**
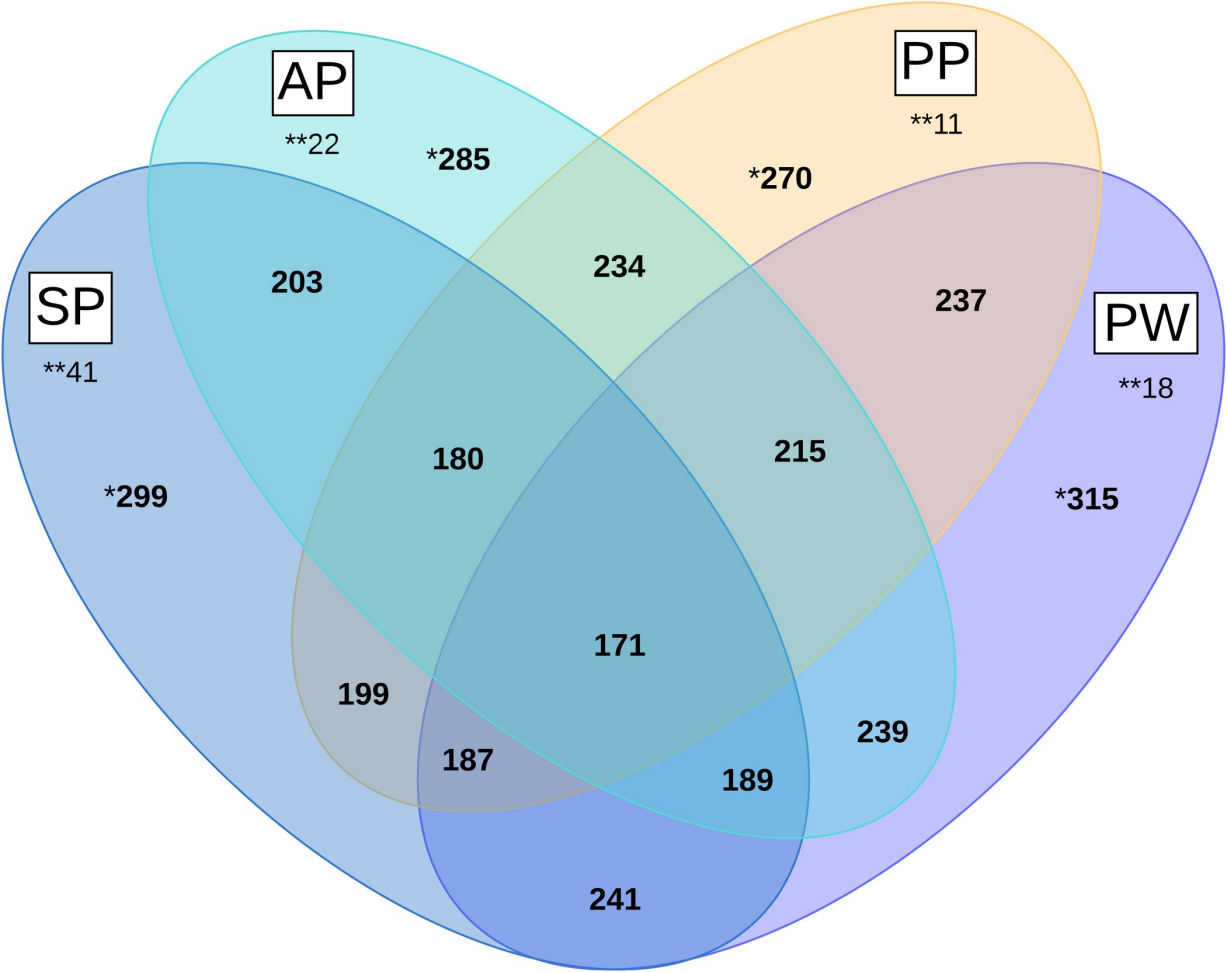
Venn diagram of genus distribution between different groups of animals. AP = Sows antepartum, PP = sows postpartum, SP = suckling piglets, PW = post-weaning piglets, *Total number of genera; **Number of unshared genera.

The shared number of genera between the four examined animal groups were calculated using the Software Microsoft Excel 2016 (Microsoft Corporation, Santa Rosa, California, USA) and Fig 1 was drawn up with the online software Lucidchart (Lucid Software Inc. 2020, Utah, USA,).

### Nonmetric multidimensional scaling (NMDS) and hierarchical clustering

Nonmetric Multidimensional Scaling (NMDS) was opted as an ordination method to visualize genus level data using the R package “Vegan”. Counts were normalized using Trimmed Mean of M-values method (TMM) [26], implemented in R package “EdgeR” with a trimming threshold of 30% [26]. The matrix of pairwise dissimilarities between samples was calculated based on Bray-Curtis dissimilarity measure [27]. Hierarchical clustering was applied to detect clusters among observations. Agglomeration on each step was performed with Ward linkage method [28]. Optimal number of clusters was chosen based on Silhouette coefficient [29].

To account for information about the close relationship between mother and its offspring, a new “family unit” was generated. Since the link between sampled sows and piglets was followed, this was exploited, and the sow-piglet ’family’ was introduced as a new unit of analysis. To obtain each family unit, three vectors of microbiome samples were pooled together: a vector of microbiome counts from a sow postpartum (PP), a vector of microbiome counts from the suckling piglet (SP) born from that sow, and a vector of microbiome counts from the same piglet post-weaning (PW). Before stacking together, each microbiome sample was normalized with TMM procedure as described above. Thus, a long vector was obtained containing the information about the microbiome composition of a sow sampled after birth and its piglet sampled after birth and after weaning. This long vector is referred to as a family unit. The aim of this approach is to neglect the differences between samples explained by individual animal characteristics and to capture the part of the variation in microbiota that can be explained by some metafactors such as farm affiliation.

A more detailed statistical protocol on NMDS and hierarchical clustering methods is described in the supporting information (S1 Protocol).

Family relations of sows and piglets, vectors containing information about genera counts of animals related to one family were stacked together. Thus, the resulting vector associated with each family unit contains information about the microbiome composition of a sow sampled before farrowing and its piglet sampled after birth and after weaning.

### Differential abundance analysis

Differential abundance analysis was performed with “*DESeq2”* package [30] on the genus level separately for sows and piglets to detect differences in microbiome composition in animals sampled at two production stages. Significance of log-fold changes was tested with Wald test and the correction for multiple testing was performed with the Benjamini-Hochberg procedure [31]. For sows, the antepartum group and for piglets the suckling piglet group was set as reference, which shows changes in the microbial community after birth and weaning. For both tested groups, the significance threshold was chosen to be 0.01 after Benjamini-Hochberg correction. A more detailed protocol of differential abundance analysis is described in the supporting information (S2 Protocol).

## Results

### Quantitative analysis of the fecal microbiota of sows and piglets

#### Comparative analysis

For the quantitative analysis of the bacterial composition of the feces samples, a total of 1.7 x 10^7^ quality combined sequence reads (19,405 ± 5635/ sample) of 802 samples with a mean read length of 284 nucleotides were used. The taxonomic assignment of all sequences revealed a total of 20 phyla, 53 orders, and 416 genera. A comprehensive overview of the relative abundance data is given in the supporting information (S1–S6 Tables). At the genus level, only dominant genera (>1%) and those detected in at least 5% of the examined samples are listed.

#### Phylum level

At the phylum level, Firmicutes (86%) followed by Bacteroidetes (11%) and Actinobacteria (1%) heavily dominated the sow feces samples before and after birth. A significant reduction of relative abundance from before to after birth was observed for 5 out of 19 phyla. Bacteroidetes and Actinobacteria showed the highest significant decrease in abundance, while Firmicutes and Proteobacteria increased significantly after birth. Furthermore, a trend for increased relative abundance of Fusobacteria, and Tenericutes was seen in sows after birth. In piglet samples, Firmicutes had a significantly lower and Actinobacteria a significantly higher relative abundance compared to sows. Nevertheless, together with Bacteriodetes these three phyla also dominated in piglet samples. Additionally, the phyla Proteobacteria, Fusobacteria, Verrucomicrobia, Spirochaetes, and Tenericutes were dominating in piglet samples with mean relative abundances >1%. Firmicutes significantly increased their abundance after weaning, while Actinobacteria and Proteobacteria displayed a significant decrease in abundance. Tenericutes and Fusobacteria showed a trend for decreased relative abundance after weaning. The phylum Chlamydiae was detected in piglets, but not in sows.

#### Order level

*Clostridiales* followed by *Lactobacillale*s and *Bacteroidales* dominated in all samples, but *Clostridiales* showed a lower relative abundance in piglet samples compared to sow samples. In sow samples, relative abundance of *Clostridiales*, *Betaproteobacteriales*, *Coriobacteriales* and *Erysiperlotrichales* increased significantly after birth. Overall, ten different orders significantly decreased their abundance after birth in sow samples. Most notably, a significant decrease was observed for the dominating *Bacteroidales*, *Lactobacillales* as well as for *Selenomonadales*. The order *Micromonosporales*, *Pasteurellales*, *and Streptomycetales* were only detected in sow samples after birth.

In piglet samples, *Clostridiales*, *Erysipelotrichales*, *Selenomonadales*, and *Bradymonadales* significantly increased their abundance after weaning. The highest decrease was seen for *Lactobacillales* and *Enterobacteriales*. A total of ten orders were only detected in post-weaning piglets. Generally, only dominant orders showed significant changes between before and after weaning. Furthermore, the number of significantly changed orders was less pronounced in piglets than in sows.

#### Genus level

In sow samples, genera of the *Clostridiales* order (*Clostridium sensu stricto* 1, *Terrisporobacter* spp., *Romboutsia* spp.), the *Bacteroidales* (unknown *Bacteroidales* BS11 gut group) and the *Lactobacilliales* (*Lactobacillus* spp., *Streptococcus* spp.) were highly dominant before and after birth (>4% abundance). Significant increases in abundance were observed for *Christensenellaceae* R-7 group, *Clostridium sensu stricto* 1, *Terrisporobacter spp*., and *Turicibacter spp*., while significantly lower abundances after birth were noted for ten other genera, most notably for the dominant *Lactobacillus spp*., *Blautia* spp., *Intestinibacter* spp. as well as for some ASV belonging to the *Lachnospiraceae*. Some genera were only detected before (*Alloprevotella* spp. and *Holdemanella* spp.) or after birth (*Lachnospiraceae* ND3007 group).

Piglet samples were heavily dominated by *Lactobacillus* spp. followed by *Bacteroides* spp., *Bifidobacterium* spp. and *Clostridium sensu stricto* 1. After weaning *Blautia* spp., *Christensenellaceae* R-7 group, *Clostridium sensu stricto* 1 and an *unknown Lachnospiraceae* significantly increased their relative abundance. The most drastic decrease in abundance after weaning was noted for *Bacteroides* spp. and *Lactobacillus* spp.. In total, nine dominant genera showed significantly reduced abundances after weaning. Ten genera were only detected as dominant in post-weaning piglets, while one genus (*Clostridium sensu stricto* 2) was only detected in samples from suckling piglets.

#### Differential abundance analysis of the fecal microbiota of ante- and postpartum sows as well as suckling and post-weaning piglets

The most pronounced quantitative changes for sows and piglets were calculated with the differential abundance analysis. The top 10 log2 fold-changes for increased as well as decreased abundances are shown in Table 1.

**Table 1:** Log2 fold-change of bacterial genera in ante- and postpartum sows as well as suckling and post-weaning piglets.

**Sow**	**Mean relative abundance antepartum** *	**Mean relative abundance postpartum**	**Log2 fold- change**
unknown *Christensenellaceae*	0.165	0.797	2.87
*Clostridiales vadin* BB60 group	0.115	0.093	2.25
*Clostridium sensu stricto* 13	0.114	0.102	2.14
unknown *Bacteroidales F082*	0.217	0.629	1.79
unknown *Clostridiales*	0.128	0.217	1.48
*Turicibacter* spp.	2.379	7.172	1.32
*Romboutsia* spp.	4.039	8.771	0.80
*Terrisporobacter* spp.	8.585	12.512	0.12
*Enterorhabdus* spp.	0.116	0.039	-4.27
*Lachnospiraceae* UCG-006	0.092	0.018	-4.30
*Fusicatenibacter* spp.	0.186	0.089	-4.51
*Faecalibacterium* spp.	1.057	0.219	-4.61
*Dialister* spp.	0.692	0.114	-4.77
*Tyzzerella* 3	0.220	n.d.	-4.81
*Subdoligranulum* spp.	1.719	0.268	-4.86
*Lachnospira* spp.	0.313	0.093	-4.91
*Coprococcus* 2	0.297	0.108	-5.83
*Streptococcus* spp.	5.416	0.712	-9.29
**Piglet**	**Mean relative abundance suckling phase** *	**Mean relative abundance post-weaning**	**Log2 fold-change**
*Prevotella* 9	0.250	3.681	7.94
*Agathobacter* spp.	0.076	2.962	7.79
*Faecalibacterium* spp.	0.187	1.530	6.39
*Lachnospiraceae* NK4A136 group	0.536	1.773	6.37
*Ruminococcaceae* UCG-008	0.096	1.074	6.10
*Catenibacterium* spp.	0.154	1.682	6.04
*Lachnospiraceae* ND3007 group	n.d.	1.309	5.95
*Lachnospiraceae* XPB1014 group	0.035	0.869	5.79
*Ruminococcus* 1	0.168	0.982	5.74
*Fusicatenibacter* spp.	0.106	1.002	5.62
*Peptostreptococcus* spp.	0.859	3.910	-4.28
*Tyzzerella* 4	0.534	n.d.	-4.29
*Butyricimonas* spp.	0.607	0.120	-4.31
*Streptococcus* spp.	3.203	0.607	-4.41
*Bacteroides* spp.	10.052	1.000	-4.59
*Enterococcus* spp.	2.468	0.910	-5.09
*Fusobacterium* spp.	3.101	2.942	-5.51
*Bifidobacterium* spp.	3.094	1.687	-5.53
*Clostridium sensu stricto* 2	2.206	0.509	-7.08
*Actinomyces* spp.	2.668	0.304	-7.09

^1^ = not detected

*reference group.

The most striking changes in abundance in sows after birth occurred for previously low abundant genera (unknown *Christensenellaceae*, unknown *Clostridiales vadin* BB60 group and *Clostridium sensu stricto* 13). However, some genera that were already dominant before birth further increased their abundance after birth (*Terrisporobacter* spp., *Romboutsia* spp. *Turicibacter* spp.). Regarding previously more dominant genera, only *Streptococcus* spp., *Subdoligranulum* spp., and *Faecalibacterium* spp. showed a drastic decrease in abundance. Interestingly, the most significant reductions in abundance occurred to already subdominant genera.

In piglets, similar changes before and after weaning occurred for genera that increased their abundance. Thus, mostly non-dominant genera like *Agathobacter* spp., *Prevotella 9* and *Faecalibacterium* spp. considerably increased their abundance to become dominant genera. In contrast to sows, a decline in abundance was observed for genera that were dominant before weaning (*Bacteroides* spp., *Streptococcus* spp. and *Fusobacterium* spp., *Enterococcus* spp., *Bifidobacterium* spp., *Actinomyces* spp., and *Clostridium sensu stricto* 2).

### Qualitative analysis of the fecal microbiota of sows and piglets

#### Genus distribution between different groups of animals

A total of 416 genera were detected in this study. Fig 1 shows a Venn diagram of the number of shared genera between four groups: sows antepartum (AP), sows postpartum (PP), suckling piglets (SP), and piglets post-weaning (PW). A core microbiota of 171 or 41% of all genera was found in all samples. Post-weaning piglets showed the highest number of genera (315), while the lowest number of genera was determined in sows postpartum (270). Differences between sows before (285 different genera) and after birth as well as between pre- (270 different genera) and post-weaning piglets (315 different genera) were only marginal. Pre- and post-weaning piglets showed the highest number of shared genera (241). Interestingly, sows and post-weaning piglets (215 different genera) share nearly 10% more genera than sows with suckling piglets (180 different genera). The number of shared genera between all piglets and sows antepartum (189) is nearly the same as the number shared between all piglets and sows postpartum (187). The most unshared genera were observed in suckling piglets and the lowest number in postpartum sows.

#### Microbiota diversity

Table 2 shows diversity indices on the ASV level of the different animal and age groups. While sows displayed a significant reduction in diversity after birth, a different outcome was visible for piglets, as number of dominant genera, as well as diversity, drastically increased after weaning. The changes in Evenness and Shannon index were similar in all groups.

**Table 2:** Diversity indices of the microbiota in sows and piglets at different time points.

Animal	Richness	Shannon	Evenness
**Sow**			
Antepartum	156 ± 58 ^b^	3.61 ± 0.57 ^b^	0.724 ± 0.083^b^
Postpartum	132 ± 45 ^a^	3.19 ± 0.42 ^a^	0.661 ± 0.065 ^a^
**Piglet**			
Suckling piglets	99 ± 26^a^	3.39 ± 0.38 ^a^	0.743 ± 0.065 ^a^
Post-weaning	159 ± 60 ^b^	3.91 ± 0.62 ^b^	0.783 ± 0.08 ^b^

^a,b^ denotes significant difference for animal type (p≤0.05), Mann-Whitney Test.

#### NMDS and hierarchical clustering

**Fig 2:**
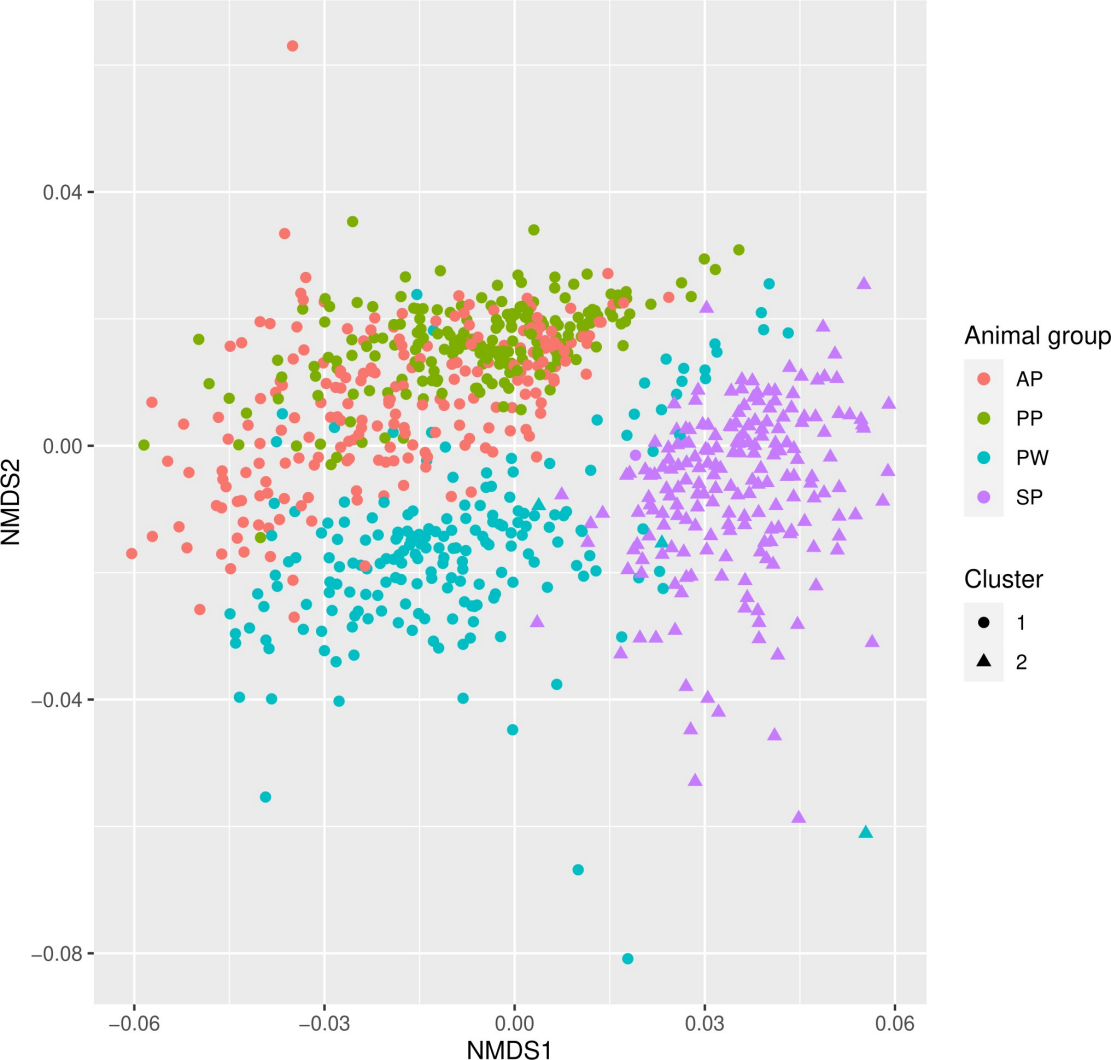
NMDS and hierarchical clustering of different animal groups. AP = sows antepartum, PP = sows postpartum, SP = suckling piglets, PW = post-weaning piglets. Filled circles and filled triangles visualize different cluster formation according to the optimal cluster formation method (S1 Fig).

The NMDS analysis of different animal groups showed a distinct formation of clusters (Fig 2). Differences between ante- and postpartum sows were less evident than for piglet data, which formed very different pre- and post-weaning clusters. Visually, there is a clear distinction between antepartum sows and post- weaning piglets. Both groups have a broadly distributed formation. The postpartum sows are located within the antepartum sows but are less broad distributed.

#### NMDS and hierarchical clustering on “family units” at genus level

**Fig 3:**
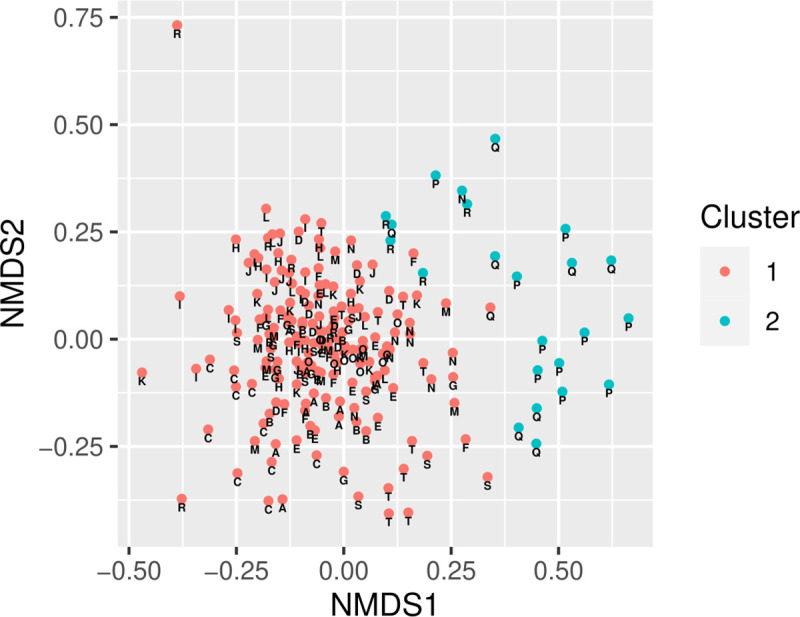
NMDS and hierarchical clustering on family units. Each data point visualizes connected data of the sampling time points PP, SP and PW for each sow and its piglet in each farm. A–T = Individual farms. Different colors visualize cluster formation according to the optimal cluster formation method (S2 Fig).

To describe the mother-child relationship in individual farms, a new unit of observation was included in the analysis, which describes the relation of one sow with one of its piglets. Thus, 10 (9 for farm A, H, and Q, respectively) family units were calculated per farm. The NMDS analysis of this dataset shows some differentiation for individual farms (Fig 3). Most notably, family units of farm P and Q formed a separate cluster. In comparison, NMDS analysis of sampling time points for sows and piglets separately did not show the clear clustering that was observed for family units (S4–S6 Figs). Overall, visual inspection shows that most family units from each farm clustered more closely together, but some individual outlier existed for many farms.

## Discussion

This study is part of the larger project Optibiom that aims to combine farm metadata and microbial composition to elucidate possible interventions to increase health in specific farms. Thus, we compared the fecal microbial composition in sows and their piglets in 20 commercial piglet production and rearing farms in Germany. The aim was to create bacterial fingerprints for different stages of production as a first step that may help to develop health strategies when combined with farm-specific metadata like feeding, husbandry, antibiotic use, and other relevant metadata.

One limit of such a large study (over 800 samples) is the sampling scheme. As farms could not be asked to change production cycles for this study, time gaps between farrowing dates and birth dates of the piglets were inevitable. Therefore, a rather long sampling period of nine months was necessary to collect a sufficient number of samples.

Overall, differences between the sow fecal microbiota before and after birth in all 20 farms showed rather drastic changes in the bacterial composition based on quantitative comparison as well as NMDS cluster formation. It is known that the microbiota of pregnant sows shifts over time [18] and that birth itself imposes stress on the animal and can lead to a modified microbiota [17]. In this study, significant changes at the genus level occurred mainly for non-dominant genera (<1% of sequences) that either increased or decreased their relative abundance. Notable exceptions were observed for the Firmicutes genera *Clostridium sensu stricto* 1, *Romboutsia* spp., *Turicibacter* spp., and *Terrisporobacter* spp. Together, these genera combined for over 60% of all sequences in postpartum sow samples. This explains the significant increase in the phylum Firmicutes after birth. Except for *Terrisporobacter* spp., these genera are known for their excellent capacity to break down carbohydrates [32–34]. Diets for lactating sows contain a high concentration of highly digestible carbohydrates and protein, most often in the form of soybean or corn meal. Undigested carbohydrates from these feed components will be fermented in the hindgut and thus one can speculate that the above-mentioned carbohydrate fermenting bacteria gained an additional advantage, as they were already dominant before birth. Their position within the microbiota will therefore further increase, possibly at the expense of *Bacteroidetes* bacteria. A similar effect on the reduction of *Bacteroidetes* has been shown in a study comparing the effect of wheat bran, a highly fermentable carbohydrate, in pregnant sows [35].

Contrary to the increase of a few members of the Firmicutes, the significant decrease of the Bacteroidetes after birth extended to genera that were not recognized as dominant before birth, except for *Alloprevotella* spp. and ASV belonging to the *Prevotellaceae*. Thus, the response of the otherwise dominating Bacteroidetes was much more diverse. The increased dominance of the few major Firmicutes genera consequently led to an overall lower bacterial diversity in postpartum sow samples.

A different trend was observed for the comparison of the microbiota development in suckling and post-weaning piglets. Here, suckling piglets displayed a much lower bacterial diversity than post-weaning piglets. This effect is part of the natural development of the microbiota in pigs [36–38] and other animal species that always show a long-term increase in diversity. Consequently, non-dominant bacteria that were probably present as contaminants from the maternal microbiota enhanced their relative abundance, while some previously dominant bacteria experienced a sharp drop in abundance. Interestingly, although the abundance of the genus *Bacteroides* spp. drastically decreased after weaning, no significant differences were observed for the *Bacteroidales* or the *Bacteroidetes*. As no other dominant *Bacteroidetes* genera were present in suckling piglets, the increase of non-dominant Bacteroidetes may reflect the increased diversity that was also observed for the increase in diversity indices.

The significant decrease in abundance of the lactic acid bacteria (*Lactobacilli*, *Streptococci*, *Enterococci*, *Bifidobacteria*) is a direct response to the change in diet. Lactic acid bacteria quickly become dominant especially in the small intestine of suckling piglets [36], as nutrient-rich components in milk, especially lactose, are readily available for fermentation. In contrast, post-weaning piglets consume solid feed with high amounts of non-starch-polysaccharides (NSP) that are not easily fermented. Consequently, NSP fermenting bacteria increase after weaning.

A core microbiota of 171 genera was observed in all animal groups; this represents 41% of all genera found. This is a lower proportion than that determined in previous studies [20, 38]. The study by Kim et al. [38] detected more commonalities between sows and post-weaning piglets than between sows and suckling piglets based on OTU analyses. This was also found in the present study based on genus level analysis. In addition, the number of unshared genera was found to be the highest in suckling piglets. This seems to be a temporary condition as the microbiota is still in development and the diet is milk-based, consistent with the lower richness of ASV. After a certain stabilization, weaned piglets and sows harbor a higher number of shared genera. This transient position of suckling piglets is also reflected in the NMDS analysis, where samples of suckling piglets are clearly contained in a separate cluster (Fig 2).

In conclusion, the shift in microbiota composition observed in sows and piglets followed a general trend that has been observed in other microbiota studies on this topic [17–20, 39], but there are a few distinguishing aspects of the present study. The animal microbiota of the different farms shows some degree of variability, which is expected, as practical conditions such as environment, antibiotic use, feeding, and management have an impact on the microbiota [21, 36, 40]. However, the differences with other studies may be due to the different methods used alone, as the comparability of microbiome studies is known to be very low [41].

Overall, the numerical trends in relative abundance are mostly consistent with the log2 fold-change trends. Not all top10 log2 fold-changes are also classified as significant changes by the Mann-Whitney test. For instance, *Fusicatenibacter* spp., ASV of *Lachnospiraceae* NK4A136 group in piglets, and ASV of Lachnospiraceae UCG-006 in sows show significant changes between the different time points according to the differential abundance analysis of log2 fold-change, but all are considered non-significant by the Mann-Whitney test. The differences in ASV of the *Clostridiales* vadin BB60 group and the genus *Clostridium sensu stricto* 13 in sows are even more apparent. Both show numerically lower abundance after birth, but according to their log2 fold-change, they have increased significantly. In piglets, the situation is similar for *Peptostreptococcus* spp. It increased numerically after weaning compared to the suckling period but decreased significantly according to log2 fold-change. Such biases have been described before for compositional data [15]. Thus, the differential abundance method seems to be the more accurate one also based on our data. Finally, the log2 fold-changes for sows and piglets did not follow the same trend. This points to the different microbiota composition between sow and piglet. While the sow contains a more stable microbiota, which is perturbed by the birth experience, the developing microbiota in piglets encounters a drastic impact due to weaning.

Despite the differences, there seem to be some consistent patterns in microbiota changes in sows before and after birth as well as in suckling and post-weaning piglets, respectively. However, these overall changes may mask individual, farm-specific differences. The first premise of the study was that the microbiota in sows and piglets hold farm-specific differences, which will be important to define future tailored strategies for individual farms. This premise is closely tied to our second premise that acknowledges the intimate association between sow and their offspring regarding microbiota development in the piglets. This hypothesis has been shown to be valid in other studies [20, 42] and also in human studies [43, 44], although there are contradictory findings in the literature for pigs [45, 46] as well. Due to the close contact of the mother with their suckling piglets and given sanitary conditions, feces represent an important vector for the transfer of the maternal microbiota [2, 3]. In fact, the introduction of the novel “family unit” to describe the mother-child ecosystem showed, although not deduced by mathematical analysis, that some farms formed a separate cluster that did not converge with the majority of farms examined. This implies that there are farm-specific differences in microbiota composition that defy the idea of an applicable generalized health concept, which may indicate that the mother-child relation is also dependent on individual conditions on each farm.

## Conclusions

The intestinal microbiota changes significantly during the production period in sows and piglets. Cluster-specific or, in some cases, even farm-specific microbiomes of sows and piglets indicate that the relationship between sows and piglets can also be specific at the microbiota level and within a farm. This leads to the assumption that generalized health maintenance strategies influencing the microbiota will have limited success under these conditions. For a deeper interpretation of these differences between farms, animals, and production times, a comprehensive analysis of the feeding, housing, and management measures of the different farms will be investigated in further studies.

## Supporting information

S1 Protocol Additional statistical information on NMDS and hierarchical clustering methods. (PDF)

S2 Protocol Additional information on differential abundance analysis. (PDF)

S1 Table Mean relative abundance at phylum level in sows at different time points. (PDF)

S2 Table Mean relative abundance at phylum level in piglets at different time points. (PDF)

S3 Table Mean relative abundance at order level in sows at different time points. (PDF)

S4 Table Mean relative abundance at order level in piglets at different time points. (PDF)

S5 Table Mean relative abundance of dominant genera (samples >1%) in sows at different time points that were detected in >5% of samples. (PDF)

S6 Table Mean relative abundance of dominant genera (samples >1%) in piglets at different time points that were detected in >5% of samples. (PDF)

S1 Fig Silhouette plot for hierarchical clustering of different animal groups. Optimal number of clusters (dashed line) for different animal groups from microbiome data of sows and piglets.
(TIF)

S2 Fig Silhouette plot for hierarchical clustering of family units. Optimal number of clusters (dashed line) for obtained family units from microbiome data of sows and their piglets at different production time points.
(TIF)

S3 Fig NMDS and hierarchical clustering on sow units. Each data point visualizes connected data of the sampling time points AP and PP for each sow in each farm. A–T = Individual farms. Different colors visualize cluster formation according to the optimal cluster formation method (S4 Fig).
(TIF)

S4 Fig Silhouette plot for hierarchical clustering of sow units. Optimal number of clusters (dashed line) for obtained sow units (AP and PP) from microbiome data of sows each farm.
(TIF)

S5 Fig NMDS and hierarchical clustering on piglet units. Each data point visualizes connected data of the sampling time points SP and PW for each piglet in each farm. A–T = Individual farms. Different colors visualize cluster formation according to the optimal cluster formation method (S6 Fig).
(TIF)

S6 Fig Silhouette plot for hierarchical clustering of piglet units. Optimal number of clusters (dashed line) for obtained piglet units (SP and PW) from microbiome data of piglets each farm.
(TIF)
