# Supplementary material for: Characterization of the fecal microbiota of sows and their offspring from German commercial pig farms
Source: PLoS One. 2021 Aug 16;16(8):e0256112. doi: 10.1371/journal.pone.0256112 (PMC8367078; doi:10.1371/journal.pone.0256112)
Supplement: S1 Protocol — (PDF) [file pone.0256112.s001.pdf]

S1 Protocol. Additional statistical information on NMDS and hierarchical clustering methods

R package "EdgeR" and implemented normalization by Trimmed Mean of M-values method

This package was used to obtain normalization factors for each microbiome sample as well as for the final estimations of transformed counts. To perform TMM normalization, a reference sample must be chosen. The default choice is a sample, upper quantile of which is the closest to the mean upper quantile over all samples. Normalization factor for sample j, given that sample r as a reference library, is calculated as follows:

$$\log_2(TMM_k^{(r)}) = \frac{\sum_{i \in I^*} w_{ij}^r M_{ij}^r}{\sum_{i \in I^*} w_{ij}^r}$$

where

- $M_{ij}^r = \log_2 \frac{K_{ij}/m_j}{K_{ir}/m_r}$ is log-fold change of genus i in sample j compared to reference sample r
- $w_{ij}^r = \frac{m_j - K_{ij}}{m_j K_{ij}} + \frac{m_r - K_{ir}}{m_r K_{ir}}$ are the weights, calculated as the inverse of the asymptotic variance calculated by delta method [1]. By entering weights into equation, TMM normalization accounts for genera variability. The more variance is observed in the counts of certain genus, the less contribution it makes.
- $K_{ij}, K_{ir} > 0$ are counts for i^{th} genus in samples j and r correspondingly
- I^* represents the collection of all genera which was not trimmed based on their M-values

The value of normalization factor depends on I^*, a collection of genera which were selected after the procedure of trimming. Specifically, upper, and lower $x\%$-values of M_{ij}^r values are trimmed. By default, in EdgeR, this trimming threshold is set to 30%.

Bray-Curtis dissimilarity measure

The Bray-Curtis dissimilarity measure is given as follows:

$$BC_{ij} = 1 - \frac{2C_{ij}}{S_i + S_j}$$

where

- C_{ij} is the sum of the smallest count of genera found in both samples
- S_i and S_j are total numbers of genera found in sample i and j correspondingly
- $BC_{ij} \in [0,1]$

At the following step, Bray-Curtis dissimilarity matrix was used for agglomerative hierarchical clustering.

Silhouette coefficient for hierarchical clustering

Silhouette coefficient considers how well each object fits into the cluster it was classified compared to all other clusters. Silhouette value for observation i is given by:

$$s(i) = \frac{b(i) - a(i)}{max(a(i), b(i))}$$

where

- $a(i) = \frac{1}{|C_i|-1} \Sigma_{j \in C_i, i \neq j} d(i,j)$ is the average distance between observation i and all other observations from cluster C_i
- $b(i) = \underset{k \neq i}{min} \frac{1}{|C_k|} \Sigma_{j \in C_k} d(i,j)$ is the smallest average dissimilarity of observation i to the member of the cluster C_k, which i does not belong to
- $-1 \leq s(i) \leq 1$
- $|C_i|$ is the size of i-th cluster

The final silhouette coefficient for a given cluster division in a data set is given by its maximum average value of silhouette measure over all observations [2]:

$$SC = \underset{k}{max} \tilde{s}(k)$$

where

- $\tilde{s}$ is an average of silhouette coefficients calculated for all samples in a data set
- k is a number of clusters in a given setting

References

1. Casella, George, Berger Roger L. (2020) Statistical Inference. Pacific Grove, CA: Duxbury Press
2. Kaufman, Leonard and Rousseeuw, Peter J. (1990) Finding groups in data: An introduction to cluster analysis. Hoboken, NJ: Wiley-Interscience
