# Supplementary material for: Characterization of the fecal microbiota of sows and their offspring from German commercial pig farms
Source: PLoS One. 2021 Aug 16;16(8):e0256112. doi: 10.1371/journal.pone.0256112 (PMC8367078; doi:10.1371/journal.pone.0256112)
Supplement: S2 Protocol — (PDF) [file pone.0256112.s002.pdf]

S2 Protocol. Additional information on Differential abundance analysis

The differential abundance analysis was performed with "DESeq2" package. "DESeq2" models count data with negative binomial distribution:

$$K_{ij} \sim NB(\mu_{ij}, \alpha_i)$$

$$\mu_{ij} = s_j q_{ij}$$

and

$$\log_2(q_{ij}) = x_{j.}\beta_i$$

where:

- K_{ij} is a count for genus i in sample j
- α_i is a genus-specific dispersion parameter
- s_j is a sample-specific normalization factor (also called a size factor) which accounts for library depth
- q_{ij} is a parameter proportional to the expected true genera abundance in sample j
