# Supplementary material for: Characterization of the fecal microbiota of sows and their offspring from German commercial pig farms
Source: PLoS One. 2021 Aug 16;16(8):e0256112. doi: 10.1371/journal.pone.0256112 (PMC8367078; doi:10.1371/journal.pone.0256112)
Supplement: S1 Table — (PDF) [file pone.0256112.s003.pdf]

S1 Table. Mean relative abundance at phylum level in sows at different time points.

Time points	Antepartum			Postpartum				Total		
	Mean	SD	SEM	Mean	SD	SEM	p-value	Mean	SD	SEM
Actinobacteria	1.183[b]	2.056	0.145	0.787[a]	2.412	0.179	<0.001	0.996	2.237	0.114
Bacteroidetes	13.324[b]	11.652	0.812	8.662[a]	12.602	0.893	<0.001	11.033	12.336	0.613
Deferribacteres	0.047	n.a.	n.a.	0.045	0.021	0.015	1.000	0.046	0.015	0.009
Elusimicrobia	0.068	0.078	0.026	0.039	0.049	0.015	0.119	0.052	0.064	0.014
Epsilonbacteraeota	0.093[b]	0.065	0.009	0.066[a]	0.066	0.011	0.004	0.082	0.066	0.007
Fibrobacteres	0.118	0.099	0.012	0.132	0.158	0.019	0.956	0.125	0.132	0.011
Firmicutes	83.868[a]	11.792	0.822	88.760[b]	12.760	0.905	<0.001	86.272	12.504	0.621
Fusobacteria	0.066	0.014	0.010	0.195	0.184	0.061	0.239	0.172	0.173	0.052
Kiritimatiellaeota	0.185	0.229	0.021	0.272	0.866	0.076	0.285	0.231	0.648	0.041
Lentisphaerae	0.053	0.044	0.015	0.118	0.328	0.049	0.785	0.108	0.303	0.042
Patescibacteria	0.108[b]	0.165	0.020	0.065[a]	0.073	0.010	0.025	0.090	0.135	0.012
Planctomycetes	0.424	1.327	0.152	0.304	0.614	0.063	0.167	0.358	0.996	0.076
Proteobacteria	0.327[a]	0.543	0.043	0.355[b]	1.394	0.110	0.003	0.341	1.058	0.059
Spirochaetes	0.911	1.363	0.099	0.958	1.383	0.101	0.535	0.934	1.371	0.071
Synergistetes	0.116	0.092	0.026	0.171	0.322	0.043	0.827	0.161	0.293	0.035
Tenericutes	0.118	0.250	0.025	0.202	0.335	0.030	0.054	0.164	0.302	0.020
Verrucomicrobia	0.097	0.166	0.034	0.113	0.144	0.019	0.567	0.108	0.150	0.016
WPS-2	0.279	0.329	0.040	0.269	0.613	0.153	0.286	0.277	0.393	0.043

[a,b] denotes significant differences between antepartum and postpartum ($p \leq 0.05$), Mann-Whitney Test;
n.a.= not available
