# Supplementary material for: Characterization of the fecal microbiota of sows and their offspring from German commercial pig farms
Source: PLoS One. 2021 Aug 16;16(8):e0256112. doi: 10.1371/journal.pone.0256112 (PMC8367078; doi:10.1371/journal.pone.0256112)
Supplement: S2 Table — (PDF) [file pone.0256112.s004.pdf]

S2 Table. Mean relative abundance at phylum level in piglets at different time points.

Time points	Suckling period			Post-weaning				Total		
	Mean	SD	SEM	Mean	SD	SEM	p-value	Mean	SD	SEM
Actinobacteria	5.137[b]	6.384	0.455	2.782[a]	8.541	0.615	<0.001	3.972	7.611	0.385
Bacteroidetes	15.883	11.887	0.845	14.383	7.693	0.547	0.923	15.133	10.027	0.504
Chlamydiae	0.179	0.186	0.093	0.377	0.868	0.161	0.912	0.353	0.817	0.142
Deferribacteres	0.173	n.a.	n.a.	0.312	0.418	0.148	0.439	0.296	0.393	0.131
Elusimicrobia	0.676	0.981	0.566	0.037	0.016	0.006	0.197	0.250	0.586	0.195
Epsilonbacteraeota	0.433	0.799	0.092	0.552	1.264	0.131	0.701	0.498	1.080	0.083
Fibrobacteres	n.d.	n.a.	n.a.	0.121	0.144	0.040	n.a.	0.121	0.144	0.040
Firmicutes	70.542[a]	15.739	1.119	79.700[b]	12.045	0.854	<0.001	75.133	14.724	0.739
Fusobacteria	3.153	6.004	0.523	2.952	7.702	1.925	0.702	3.131	6.179	0.508
Kiritimatiellaeota	n.d.	n.a.	n.a.	0.202	0.219	0.033	n.a.	0.202	0.219	0.033
Lentisphaerae	0.287	0.479	0.092	0.066	0.053	0.014	0.394	0.211	0.401	0.063
Patescibacteria	0.027	0.008	0.006	0.140	0.185	0.030	0.086	0.134	0.182	0.029
Planctomycetes	0.292	0.379	0.077	0.232	0.269	0.044	0.565	0.256	0.315	0.040
Proteobacteria	4.524[b]	6.086	0.436	1.630[a]	3.812	0.306	<0.001	3.242	5.392	0.288
Spirochaetes	1.647	2.836	0.494	0.831	1.479	0.136	0.534	1.009	1.881	0.153
Synergistetes	0.953	1.135	0.173	1.040	1.740	0.282	0.307	0.994	1.442	0.160
Tenericutes	1.009	2.489	0.665	0.307	0.788	0.073	0.136	0.384	1.109	0.098
Verrucomicrobia	4.255	6.944	0.972	4.192	7.869	1.855	0.085	4.239	7.137	0.859
WPS-2	0.595	n.a.	n.a.	0.615	1.687	0.436	0.233	0.614	1.630	0.408

[a,b] denotes significant differences between suckling period and post-weaning ($p \leq 0.05$), Mann-Whitney Test;
n.a. = not available; n.d. = not detected
