# Supplementary material for: Characterization of the fecal microbiota of sows and their offspring from German commercial pig farms
Source: PLoS One. 2021 Aug 16;16(8):e0256112. doi: 10.1371/journal.pone.0256112 (PMC8367078; doi:10.1371/journal.pone.0256112)
Supplement: S3 Table — (PDF) [file pone.0256112.s005.pdf]

S3 Table. Mean relative abundance at order level in sows at different time points.

Time points	Antepartum			Postpartum				Total		
	Mean	SD	SEM	Mean	SD	SEM	p-value	Mean	SD	SEM
Actinomycetales	0.240	0.428	0.191	0.616	0.908	0.371	0.273	0.445	0.724	0.218
Aeromonadales	0.309[b]	0.624	0.067	0.217[a]	0.592	0.070	0.033	0.267	0.609	0.048
Anaeroplasmatales	0.031	0.025	0.009	0.033	0.018	0.007	0.749	0.032	0.020	0.005
Bacillales	0.133	0.237	0.075	0.142	0.158	0.053	0.142	0.137	0.198	0.045
Bacteroidales	13.319[b]	11.653	0.812	8.659[a]	12.602	0.893	<0.001	11.030	12.336	0.613
Betaproteobacteriales	0.164[a]	0.239	0.041	0.453[b]	2.124	0.388	0.006	0.299	1.459	0.182
Bifidobacteriales	0.989[b]	1.506	0.128	0.576[a]	0.960	0.089	0.020	0.800	1.300	0.082
Bradymonadales	0.157	0.162	0.024	0.119	0.101	0.019	0.538	0.143	0.142	0.017
Campylobacterales	0.093[b]	0.065	0.009	0.066[a]	0.066	0.011	0.004	0.082	0.066	0.007
Clostridiales	61.264[a]	15.568	1.085	70.003[b]	15.730	1.115	<0.001	65.558	16.229	0.806
Coriobacteriales	0.344[a]	0.362	0.026	0.374[b]	2.003	0.160	<0.001	0.357	1.366	0.073
Corynebacteriales	0.665[b]	3.007	0.448	0.288[a]	0.982	0.253	0.004	0.571	2.646	0.342
Deferribacterales	0.047	n.a.	n.a.	0.045	0.021	0.015	1.000	0.046	0.015	0.009
Desulfovibrionales	0.082	0.089	0.010	0.080	0.103	0.011	0.253	0.081	0.097	0.007
Elusimicrobiales	0.068	0.078	0.026	0.039	0.049	0.015	0.119	0.052	0.064	0.014
Enterobacteriales	0.121	0.203	0.031	0.290	0.701	0.093	0.055	0.217	0.548	0.055
Erysipelotrichales	2.756[a]	2.213	0.154	7.404[b]	4.935	0.350	<0.001	5.040	4.454	0.221
Fibrobacterales	0.118	0.099	0.012	0.132	0.158	0.019	0.956	0.125	0.132	0.011
Fusobacteriales	0.066	0.014	0.010	0.195	0.184	0.061	0.239	0.172	0.173	0.052
Gastranaerophilales	0.088[b]	0.077	0.009	0.053[a]	0.041	0.006	0.002	0.073	0.066	0.006
Izimaplasmatales	0.033	0.023	0.011	0.055	0.034	0.011	0.280	0.049	0.032	0.009
Kiritimatiellaeota WCHB1-41	0.185	0.229	0.021	0.272	0.866	0.076	0.285	0.231	0.648	0.041
Lactobacillales	18.800[b]	15.848	1.104	11.427[a]	12.838	0.931	<0.001	15.263	14.927	0.750
Micrococcales	0.074	0.089	0.025	0.491	1.090	0.412	0.143	0.220	0.649	0.145
Micromonosporales	n.d.	n.a.	n.a.	0.438	n.a.	n.a.	n.a.	0.438	n.a.	n.a.

Mollicutes RF39	0.122	0.261	0.028	0.200	0.341	0.032	0.206	0.166	0.311	0.022
Mycoplasmatales	0.040	0.033	0.010	0.110	0.182	0.058	0.091	0.073	0.130	0.028
Oligosphaerales	0.062	0.028	0.012	0.130	0.346	0.055	0.448	0.122	0.327	0.049
Opitutales	0.038	0.032	0.016	0.074	0.074	0.020	0.339	0.066	0.068	0.016
Paracaedibacterales	0.016	0.006	0.005	n.d.	n.a.	n.a.	n.a.	0.016	0.006	0.005
Pasteurellales	n.d.	n.a.	n.a.	0.028	n.a.	n.a.	n.a.	0.028	n.a.	n.a.
Pirellulales	0.424	1.327	0.152	0.304	0.614	0.063	0.167	0.358	0.996	0.076
Propionibacteriales	0.162[b]	0.222	0.044	0.109[a]	0.150	0.026	0.031	0.131	0.184	0.024
Pseudomonadales	0.039	0.010	0.006	n.d.	n.a.	n.a.	n.a.	0.039	0.010	0.006
Rhodospirillales	0.028	0.017	0.005	0.023	0.008	0.004	0.896	0.026	0.015	0.004
Saccharimonadales	0.108[b]	0.165	0.020	0.065[a]	0.073	0.010	0.025	0.090	0.135	0.012
Selenomonadales	1.048[b]	0.990	0.071	0.534[a]	0.963	0.079	<0.001	0.826	1.010	0.055
Spirochaetales	0.911	1.363	0.099	0.958	1.383	0.101	0.535	0.934	1.371	0.071
Streptomycetales	n.d.	n.a.	n.a.	1.296	n.a.	n.a.	n.a.	1.296	n.a.	n.a.
Synergistales	0.116	0.092	0.026	0.171	0.322	0.043	0.827	0.161	0.293	0.035
unknown Actinobacteria	0.026	n.a.	n.a.	n.d.	n.a.	n.a.	n.a.	0.026	n.a.	n.a.
unknown Bacteroidetes	0.087	0.122	0.039	0.074	0.044	0.018	0.588	0.082	0.098	0.025
unknown Firmicutes	0.144	0.344	0.039	0.102	0.100	0.011	0.880	0.123	0.251	0.020
unknown Proteobacteria	0.027	0.004	0.002	0.021	0.008	0.004	0.275	0.024	0.006	0.003
unknown WPS-2	0.279	0.329	0.040	0.269	0.613	0.153	0.286	0.277	0.393	0.043
Verrucomicrobiales	0.104	0.176	0.038	0.122	0.157	0.023	0.598	0.116	0.162	0.020
Victivallales	0.028	0.015	0.007	0.022	0.010	0.004	0.522	0.025	0.011	0.004

[a,b] denotes significant differences between antepartum and postpartum ($p \leq 0.05$), Mann-Whitney Test;
n.a.= not available
