# Supplementary material for: Characterization of the fecal microbiota of sows and their offspring from German commercial pig farms
Source: PLoS One. 2021 Aug 16;16(8):e0256112. doi: 10.1371/journal.pone.0256112 (PMC8367078; doi:10.1371/journal.pone.0256112)
Supplement: S4 Table — (PDF) [file pone.0256112.s006.pdf]

S4 Table. Mean relative abundance at order level in piglets at different time points.

Time points	Suckling period			Post-weaning				Total		
	Mean	SD	SEM	Mean	SD	SEM	p-value	Mean	SD	SEM
Actinomycetales	2.644[b]	4.077	0.348	0.576[a]	1.252	0.473	0.019	2.544	4.009	0.334
Aeromonadales	0.369	0.386	0.173	0.697	1.958	0.253	0.844	0.672	1.884	0.234
Anaeroplasmatales	n.d.	n.a.	n.a.	0.182	0.217	0.125	n.a.	0.182	0.217	0.125
Bacillales	0.501	1.491	0.292	0.143	0.027	0.019	0.656	0.476	1.438	0.272
Bacteroidales	15.877	11.885	0.845	14.381	7.689	0.546	0.931	15.129	10.025	0.504
Betaproteobacteriales	1.123	2.720	0.316	0.515	1.546	0.231	0.076	0.894	2.357	0.216
Bifidobacteriales	3.025	6.120	0.697	1.732	3.037	1.148	0.692	2.917	5.924	0.646
Bradymonadales	0.072[a]	0.046	0.019	0.631[b]	1.029	0.141	0.010	0.574	0.989	0.129
Campylobacterales	0.433	0.799	0.092	0.552	1.264	0.131	0.701	0.498	1.080	0.083
Chlamydiales	0.179	0.186	0.093	0.377	0.868	0.161	0.912	0.353	0.817	0.142
Clostridiales	36.044[a]	16.213	1.152	60.420[b]	17.405	1.234	<0.001	48.263	20.764	1.042
Coriobacteriales	1.787	2.915	0.210	2.689	8.516	0.613	0.425	2.239	6.379	0.325
Corynebacteriales	0.242	0.441	0.056	0.110	0.056	0.023	0.863	0.230	0.422	0.051
Deferribacterales	0.173	n.a.	n.a.	0.312	0.418	0.148	0.439	0.296	0.393	0.131
Desulfovibrionales	0.209[b]	0.185	0.015	0.169[a]	0.157	0.015	0.029	0.193	0.175	0.011
Elusimicrobiales	0.676	0.981	0.566	0.037	0.016	0.006	0.197	0.250	0.586	0.195
Enterobacteriales	4.049[b]	5.837	0.440	1.682[a]	4.748	0.531	<0.001	3.310	5.619	0.351
Erysipelotrichales	2.623[a]	4.438	0.321	3.066[b]	4.967	0.355	<0.001	2.848	4.712	0.240
Fibrobacterales	n.d.	n.a.	n.a.	0.121	0.144	0.040	n.a.	0.121	0.144	0.040
Flavobacteriales	n.d.	n.a.	n.a.	0.048	n.a.	n.a.	n.a.	0.048	n.a	n.a.
Fusobacteriales	3.153	6.004	0.523	2.952	7.702	1.925	0.702	3.131	6.179	0.508
Gastranaerophilales	0.073	0.044	0.025	0.176	0.387	0.060	0.927	0.169	0.375	0.056
Izimaplasmatales	0.078	0.036	0.021	0.037	0.017	0.007	0.121	0.051	0.031	0.010
Kiritimatiellaeota WCHB1-41	n.d.	n.a.	n.a.	0.202	0.219	0.033	n.a.	0.202	0.219	0.033
Lactobacillales	31.225[b]	19.121	1.362	15.131[a]	15.492	1.145	<0.001	23.474	19.214	0.986
Micrococcales	0.283	0.395	0.035	0.119	0.103	0.036	0.123	0.273	0.386	0.033

Mollicutes RF39	1.158[b]	2.670	0.771	0.306[a]	0.794	0.075	0.044	0.387	1.125	0.101
Mycoplasmatales	n.d.	n.a.	n.a.	0.050	n.a.	n.a.	n.a.	0.050	n.a	n.a.
Oligosphaerales	0.044	0.019	0.013	0.059	0.040	0.012	0.844	0.056	0.038	0.010
Opitutales	0.044	0.010	0.007	3.181	4.361	2.180	0.355	2.136	3.746	1.529
Paracaedibacterales	n.d.	n.a.	n.a.	0.103	0.005	0.003	n.a.	0.103	0.005	0.003
Pasteurellales	0.818	2.857	0.357	0.156	0.248	0.111	0.267	0.770	2.756	0.332
Pirellulales	0.292	0.379	0.077	0.232	0.269	0.044	0.565	0.256	0.315	0.040
Propionibacteriales	1.230	2.576	0.591	0.046	0.023	0.017	0.338	1.117	2.469	0.539
Pseudomonadales	0.036	0.018	0.009	0.179	n.a.	n.a.	0.157	0.065	0.066	0.029
Rhizobiales	0.108	n.a.	n.a.	n.d.	n.a.	n.a.	n.a.	0.108	n.a	n.a.
Rhodobacterales	0.186	n.a.	n.a.	n.d.	n.a.	n.a.	n.a.	0.186	n.a	n.a.
Rhodospirillales	n.d.	n.a.	n.a.	0.080	0.109	0.054	n.a.	0.080	0.109	0.054
Rickettsiales	n.d.	n.a.	n.a.	0.016	n.a.	n.a.	n.a.	0.016	n.a	n.a.
Saccharimonadales	0.027	0.008	0.006	0.140	0.185	0.030	0.086	0.134	0.182	0.029
Selenomonadales	0.851[a]	1.188	0.091	2.454[b]	2.773	0.203	<0.001	1.691	2.309	0.123
Sphingomonadales	0.062	n.a.	n.a.	0.030	n.a.	n.a.	0.317	0.046	0.022	0.016
Spirochaetales	1.647	2.836	0.494	0.831	1.479	0.136	0.534	1.009	1.881	0.153
Streptomycetales	n.d.	n.a.	n.a.	0.024	n.a.	n.a.	n.a.	0.024	n.a	n.a.
Synergistales	0.953	1.135	0.173	1.040	1.740	0.282	0.307	0.994	1.442	0.160
unknown Actinobacteria	0.018	0.001	0.001	n.d.	n.a.	n.a.	n.a.	0.018	0.001	0.001
unknown Bacteroidetes	0.304	0.284	0.142	0.160	0.160	0.093	0.724	0.242	0.234	0.089
unknown Firmicutes	0.207	0.364	0.036	0.156	0.148	0.018	0.388	0.188	0.301	0.023
unknown Proteobacteria	n.d.	n.a.	n.a.	0.124	0.208	0.104	n.a.	0.124	0.208	0.104
unknown WPS-2	0.595	n.a.	n.a.	0.615	1.687	0.436	0.233	0.614	1.630	0.408
Verrucomicrobiales	4.427[b]	7.032	1.005	4.182[a]	8.489	2.192	0.032	4.370	7.328	0.916
Victivallales	0.294	0.487	0.095	0.071	0.089	0.044	0.246	0.265	0.459	0.084

[a,b] denotes significant differences between suckling period and post-weaning ($p \leq 0.05$), Mann-Whitney Test;
n.a. = not available; n.d. = not detected
