# Supplementary material for: Characterization of the fecal microbiota of sows and their offspring from German commercial pig farms
Source: PLoS One. 2021 Aug 16;16(8):e0256112. doi: 10.1371/journal.pone.0256112 (PMC8367078; doi:10.1371/journal.pone.0256112)
Supplement: S5 Table — (PDF) [file pone.0256112.s007.pdf]

S5 Table. Mean relative abundance of dominant genera (samples >1%) in sows at different time points that were detected in >5% of samples.

Time points	Antepartum			Postpartum				Total		
	Mean	SD	SEM	Mean	SD	SEM	p-value	Mean	SD	SEM
Agathobacter	2.022	0.753	0.148	1.297	n.a.	n.a.	0.304	1.995	0.751	0.145
Alloprevotella	1.101	0.057	0.025	n.d.	n.a.	n.a.	n.a.	1.101	0.057	0.025
Bacteroides	1.424	0.117	0.083	2.498	1.579	0.499	0.519	2.319	1.488	0.430
Bifidobacterium	2.912	1.808	0.297	2.609	1.434	0.370	0.664	2.824	1.700	0.236
Blautia	2.528	1.098	0.130	1.664	0.662	0.296	0.073	2.471	1.093	0.125
Christensenellaceae R-7 group	2.639[a]	2.693	0.265	3.308[b]	3.157	0.251	0.002	3.044	2.995	0.185
Clostridiales Family XIII AD3011 group	1.446	0.488	0.106	1.451	0.554	0.109	0.966	1.449	0.520	0.076
Clostridium sensu stricto 1	26.230[a]	12.326	0.861	32.746[b]	12.786	0.906	<0.001	29.439	12.956	0.645
Clostridium sensu stricto 2	1.273	0.176	0.056	1.523	0.599	0.173	0.429	1.409	0.466	0.099
Collinsella	1.042	n.a.	n.a.	3.469	n.a.	n.a.	0.317	2.256	1.716	1.214
Coprococcus 3	1.570	0.791	0.138	1.550	0.364	0.210	0.587	1.569	0.761	0.127
Dorea	1.438	0.369	0.083	1.260	n.a.	n.a.	0.741	1.430	0.362	0.079
Escherichia/Shigella	1.324	n.a.	n.a.	2.194	1.354	0.605	0.770	2.049	1.262	0.515
Faecalibacterium	2.346	1.127	0.190	1.775	0.710	0.502	0.546	2.315	1.109	0.182
Holdemanella	1.714	n.a.	n.a.	n.d.	n.a.	n.a.	n.a.	1.714	n.a.	n.a.
Intestinibacter	2.111[b]	0.587	0.099	1.387[a]	0.134	0.094	0.037	2.072	0.594	0.098
Lachnospiraceae AC2044 group	1.645	0.584	0.162	1.622	0.648	0.229	0.664	1.636	0.593	0.129
Lachnospiraceae ND3007 group	n.d.	n.a.	n.a.	1.810	n.a.	n.a.	n.a.	1.810	n.a.	n.a.
Lachnospiraceae NK4A136 group	1.334	0.322	0.067	1.238	0.257	0.071	0.300	1.300	0.300	0.050
Lachnospiraceae UCG-007	1.637[b]	0.514	0.057	1.361[a]	0.287	0.051	0.016	1.560	0.477	0.045
Lachnospiraceae XPB1014 group	1.945	1.046	0.130	1.493	0.601	0.102	0.008	1.787	0.937	0.094
Lactobacillus	15.517[b]	12.504	0.922	13.273[a]	12.830	1.034	0.038	14.495	12.684	0.690
Marvinbryantia	1.456	0.455	0.062	1.403	0.432	0.216	0.782	1.453	0.450	0.059
Megasphaera	1.667	0.497	0.222	1.964	1.161	0.670	0.655	1.778	0.741	0.262

Phascolarctobacterium	1.501	0.367	0.106	1.097	n.a.	n.a.	0.285	1.470	0.369	0.102
Prevotella 9	4.260	2.955	0.332	2.953	2.219	0.785	0.138	4.140	2.910	0.312
Prevotellaceae NK3B31 group	2.810[b]	1.514	0.144	2.108[a]	1.019	0.159	0.011	2.620	1.428	0.116
Prevotellaceae UCG-001	1.553[a]	0.526	0.112	2.220[b]	0.963	0.182	0.009	1.927	0.861	0.122
Rikenellaceae RC9 gut group	1.661	0.626	0.081	1.615	0.629	0.068	0.564	1.634	0.626	0.052
Romboutsia	4.237[a]	2.197	0.158	8.812[b]	3.325	0.236	<0.001	6.548	3.633	0.183
Roseburia	1.597	0.562	0.230	1.791	n.a.	n.a.	0.617	1.625	0.518	0.196
Ruminococcaceae NK4A214 group	1.748	0.824	0.097	1.556	0.570	0.061	0.175	1.642	0.700	0.055
Ruminococcaceae UCG-002	1.675	0.668	0.090	1.608	0.734	0.084	0.352	1.636	0.705	0.061
Ruminococcaceae UCG-005	2.602[b]	1.245	0.104	1.882[a]	1.069	0.103	<0.001	2.292	1.223	0.077
Ruminococcaceae UCG-008	1.480	0.300	0.212	1.080	n.a.	n.a.	0.221	1.346	0.314	0.181
Ruminococcaceae UCG-014	1.709	0.835	0.171	1.893	1.124	0.265	0.799	1.788	0.961	0.148
Ruminococcus 1	1.798	1.039	0.144	1.536	0.743	0.152	0.273	1.715	0.959	0.110
Ruminococcus 2	3.157	1.977	1.398	3.089	n.a.	n.a.	1.000	3.135	1.398	0.807
Streptococcus	9.274	10.628	1.108	20.106	n.a.	n.a.	0.180	9.391	10.630	1.102
Subdoligranulum	4.465	2.779	0.397	3.499	n.a.	n.a.	0.917	4.446	2.754	0.389
Terrisporobacter	8.664[a]	3.632	0.254	12.512[b]	4.546	0.322	<0.001	10.564	4.533	0.226
Treponema 2	2.391	1.979	0.286	2.433	1.965	0.278	0.584	2.413	1.962	0.198
Turicibacter	3.383[a]	2.276	0.197	7.598[b]	4.809	0.352	<0.001	5.846	4.467	0.250
unknown Bacteroidales p-2534-18B5 gut group	2.264	1.928	0.557	2.124	1.429	0.357	0.642	2.184	1.629	0.308
unknown Bacteroidales BS11 gut group	28.919	19.280	3.520	32.680	24.539	5.232	0.643	30.510	21.514	2.983
unknown Lachnospiraceae	2.491[b]	1.369	0.113	1.792[a]	1.602	0.231	<0.001	2.318	1.458	0.105
unknown Lactobacillales	1.459	0.533	0.082	1.232	0.197	0.041	0.084	1.379	0.455	0.056
unknown Muribaculaceae	2.634	1.873	0.162	2.338	1.473	0.184	0.305	2.538	1.755	0.125
unknown Prevotellaceae	2.809	2.575	0.562	1.537	0.374	0.187	0.335	2.605	2.402	0.480
unknown Ruminococcaceae	1.665	0.624	0.073	1.582	0.659	0.085	0.184	1.628	0.639	0.055

[a,b] denotes significant differences between antepartum and postpartum ($p \leq 0.05$), Mann-Whitney Test; n.a.= not available
