# Supplementary material for: Characterization of the fecal microbiota of sows and their offspring from German commercial pig farms
Source: PLoS One. 2021 Aug 16;16(8):e0256112. doi: 10.1371/journal.pone.0256112 (PMC8367078; doi:10.1371/journal.pone.0256112)
Supplement: S6 Table — (PDF) [file pone.0256112.s008.pdf]

S6 Table. Mean relative abundance of dominant genera (samples >1%) in piglets at different time points that were detected in >5% of samples.

Time points	Suckling period			Post-weaning				Total		
	Mean	SD	SEM	Mean	SD	SEM	p-value	Mean	SD	SEM
Actinomyces	5.232	4.722	0.590	1.225	n.a.	n.a.	0.183	5.171	4.711	0.584
Agathobacter	n.d.	n.a.	n.a.	3.888	3.262	0.302	n.a.	3.888	3.262	0.302
Alloprevotella	3.407	3.227	1.613	3.166	2.308	0.296	0.743	3.180	2.342	0.291
Bacteroides	11.580[b]	10.682	0.824	3.422[a]	3.011	0.730	<0.001	10.830	10.485	0.771
Bifidobacterium	8.387	8.280	1.624	5.213	4.013	2.837	0.789	8.160	8.048	1.521
Blautia	2.981[a]	2.586	0.386	7.122[b]	7.199	0.537	<0.001	6.294	6.744	0.450
Christensenellaceae R-7 group	4.310[a]	5.396	0.813	6.282[b]	6.188	0.622	0.026	5.675	6.007	0.502
Clostridiales Family XIII AD301 group	1.266	0.256	0.115	1.898	0.923	0.138	0.109	1.834	0.898	0.127
Clostridium sensu stricto 1	8.186[a]	7.192	0.581	13.643[b]	13.493	1.179	0.029	10.703	10.901	0.647
Clostridium sensu stricto 2	4.130	3.114	0.454	n.d.	n.a.	n.a.	n.a.	4.130	3.114	0.454
Collinsella	3.620	1.957	0.336	7.962	14.081	2.255	0.325	5.940	10.543	1.234
Coprococcus 3	1.848	0.873	0.309	3.200	2.642	0.262	0.074	3.101	2.577	0.246
Dorea	2.209	1.201	0.212	2.444	1.465	0.128	0.584	2.398	1.416	0.111
Enterococcus	5.761	8.410	1.313	5.790	5.537	3.916	0.729	5.762	8.251	1.258
Escherichia/Shigella	5.961	6.420	0.601	6.519	8.464	1.995	0.389	6.037	6.700	0.583
Faecalibacterium	n.d.	n.a.	n.a.	2.890	1.776	0.211	n.a.	2.890	1.776	0.211
Fusobacterium	7.854	7.439	1.063	8.724	12.617	5.642	0.447	7.935	7.887	1.073
Holdemanella	4.119[b]	3.386	0.640	2.983[a]	3.623	0.725	0.026	3.583	3.513	0.483
Intestinibacter	n.d.	n.a.	n.a.	2.352	1.768	0.625	n.a.	2.352	1.768	0.625
Lachnoclostridium	5.048[b]	3.342	0.253	2.796[a]	2.311	0.560	0.001	4.848	3.322	0.240
Lachnospiraceae AC2044 group	1.870	0.876	0.620	2.080	1.117	0.197	0.942	2.067	1.094	0.188
Lachnospiraceae ND3007 group	n.d.	n.a.	n.a.	1.822	0.671	0.106	n.a.	1.822	0.671	0.106
Lachnospiraceae NK4A136 group	n.d.	n.a.	n.a.	2.622	1.688	0.167	n.a.	2.622	1.688	0.167
Lachnospiraceae XPB1014 group	n.d.	n.a.	n.a.	2.650	1.467	0.244	n.a.	2.650	1.467	0.244

Lactobacillus	28.489[b]	18.543	1.360	17.936[a]	15.374	1.255	<0.001	23.778	17.962	0.980
Marvinbryantia	1.587	0.610	0.193	1.659	0.582	0.072	0.559	1.649	0.582	0.067
Megasphaera	1.201	n.a.	n.a.	3.567	3.359	0.560	0.223	3.503	3.334	0.548
Parabacteroides	2.165	1.157	0.176	2.080	1.382	0.357	0.307	2.143	1.207	0.158
Phascolarcto-bacterium	2.331	1.260	0.226	2.010	1.285	0.149	0.135	2.105	1.280	0.125
Prevotella 9	n.d.	n.a.	n.a.	4.859	3.991	0.366	n.a.	4.859	3.991	0.366
Prevotellaceae NK3B31 group	1.955	0.586	0.207	4.085	4.150	0.376	0.152	3.953	4.054	0.356
Prevotellaceae UCG-001	3.019	1.502	0.568	2.714	0.906	0.641	1.000	2.951	1.347	0.449
Rikenellaceae RC9 gut group	3.446[b]	3.076	0.536	2.140[a]	3.178	0.537	<0.001	2.773	3.175	0.385
Romboutsia	3.428	3.455	0.384	2.985	1.248	0.395	0.526	3.379	3.284	0.344
Roseburia	2.416	1.811	0.484	2.484	1.435	0.153	0.508	2.475	1.482	0.147
Ruminococcaceae NK4A214 group	2.201	1.316	0.190	2.445	1.548	0.150	0.213	2.369	1.480	0.119
Ruminococcaceae UCG-002	6.039[b]	5.793	0.538	3.603[a]	3.113	0.307	<0.001	4.894	4.871	0.329
Ruminococcaceae UCG-005	3.526	4.087	0.746	3.664	2.545	0.220	0.100	3.639	2.874	0.224
Ruminococcaceae UCG-008	n.d.	n.a.	n.a.	2.022	1.113	0.139	n.a.	2.022	1.113	0.139
Ruminococcaceae UCG-014	2.701[b]	2.115	0.638	1.694[a]	0.898	0.146	0.052	1.920	1.317	0.188
Ruminococcus 1	n.d.	n.a.	n.a.	1.817	0.797	0.098	n.a.	1.817	0.797	0.098
Ruminococcus 2	4.426[b]	4.546	0.568	2.163[a]	1.334	0.272	0.001	3.809	4.057	0.433
Streptococcus	5.354	5.637	0.572	2.407	1.532	0.685	0.116	5.209	5.541	0.549
Subdoligranulum	5.386	5.420	0.879	3.761	3.157	0.287	0.165	4.149	3.865	0.306
Terrisporobacter	n.d.	n.a.	n.a.	3.567	2.192	0.262	n.a.	3.567	2.192	0.262
Treponema 2	5.170[b]	3.773	1.334	2.658[a]	2.333	0.497	0.004	3.328	2.942	0.537
Turicibacter	2.242	1.171	0.585	2.473	1.138	0.294	0.549	2.425	1.116	0.256
Unknown Bacteroidales p-2534-B5 gut group	2.823	1.139	0.509	2.945	2.064	0.533	0.513	2.914	1.848	0.413
unknown Erysipelotrichaceae	4.817	6.006	0.950	4.936	8.166	2.887	0.740	4.836	6.314	0.911
unknown Lachnospiraceae	4.690[a]	3.603	0.268	6.579[b]	3.509	0.251	<0.001	5.672	3.674	0.189
unknown Lactobacillales	1.558	0.616	0.276	1.679	0.486	0.184	0.570	1.629	0.521	0.150
unknown Muribaculaceae	5.951	6.081	0.927	4.629	4.671	0.396	0.426	4.941	5.053	0.375
unknown Prevotellaceae	3.811	5.195	1.134	2.255	2.211	0.350	0.387	2.791	3.568	0.457

unknown Ruminococcaceae	3.193	2.881	0.259	2.469	1.637	0.139	0.128	2.811	2.334	0.144

[a,b] denotes significant differences between suckling period and post-weaning ($p \leq 0.05$), Mann-Whitney Test;
n.a. = not available; n.d. = not detected
