# Supplementary figures and images for: Characterization of the fecal microbiota of sows and their offspring from German commercial pig farms
Source: PLoS One. 2021 Aug 16;16(8):e0256112. doi: 10.1371/journal.pone.0256112 (PMC8367078; doi:10.1371/journal.pone.0256112)

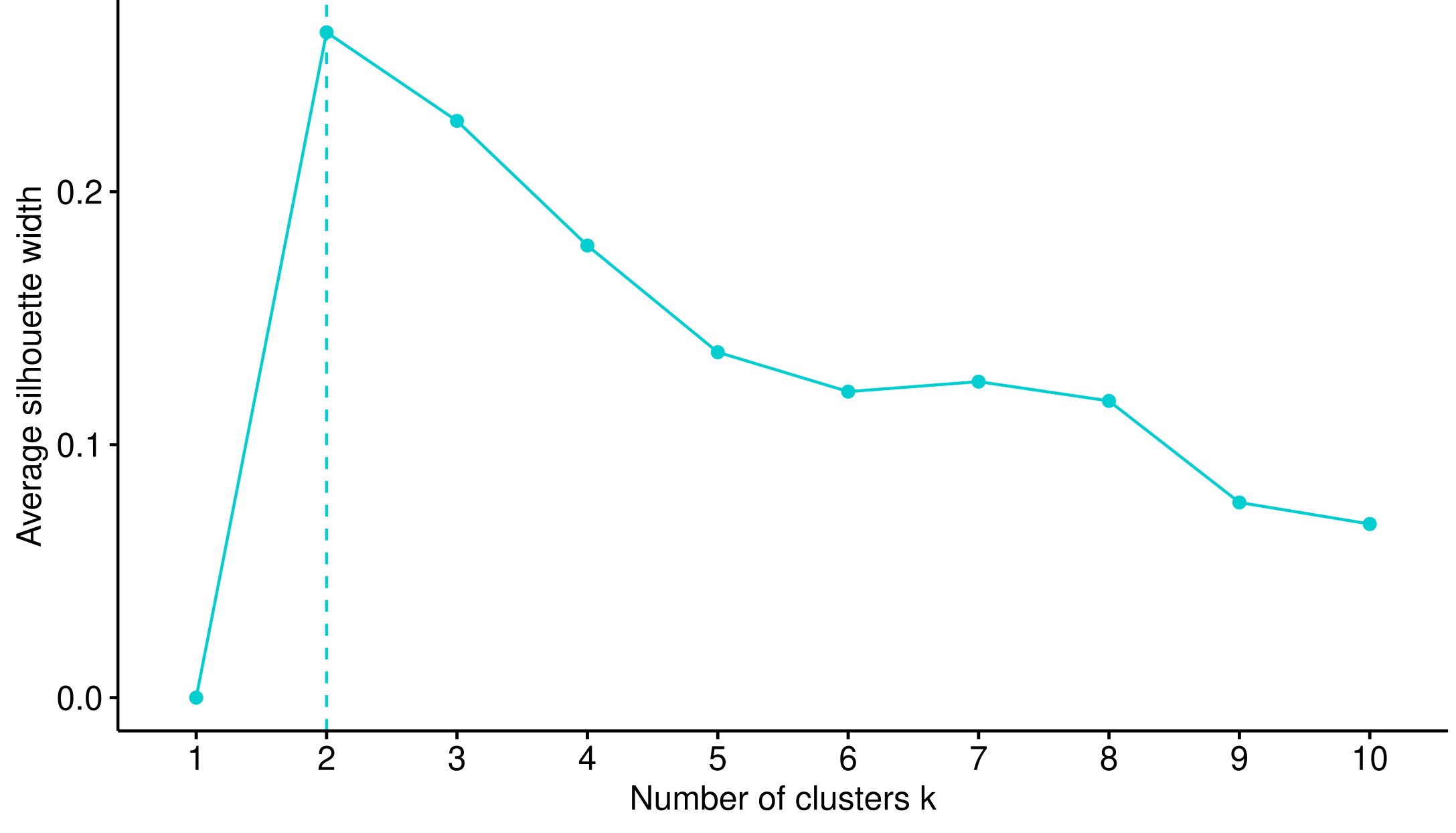

Supplement: S1 Fig — Optimal number of clusters (dashed line) for different animal groups from microbiome data of sows and piglets. (TIF) [file pone.0256112.s009.tif]

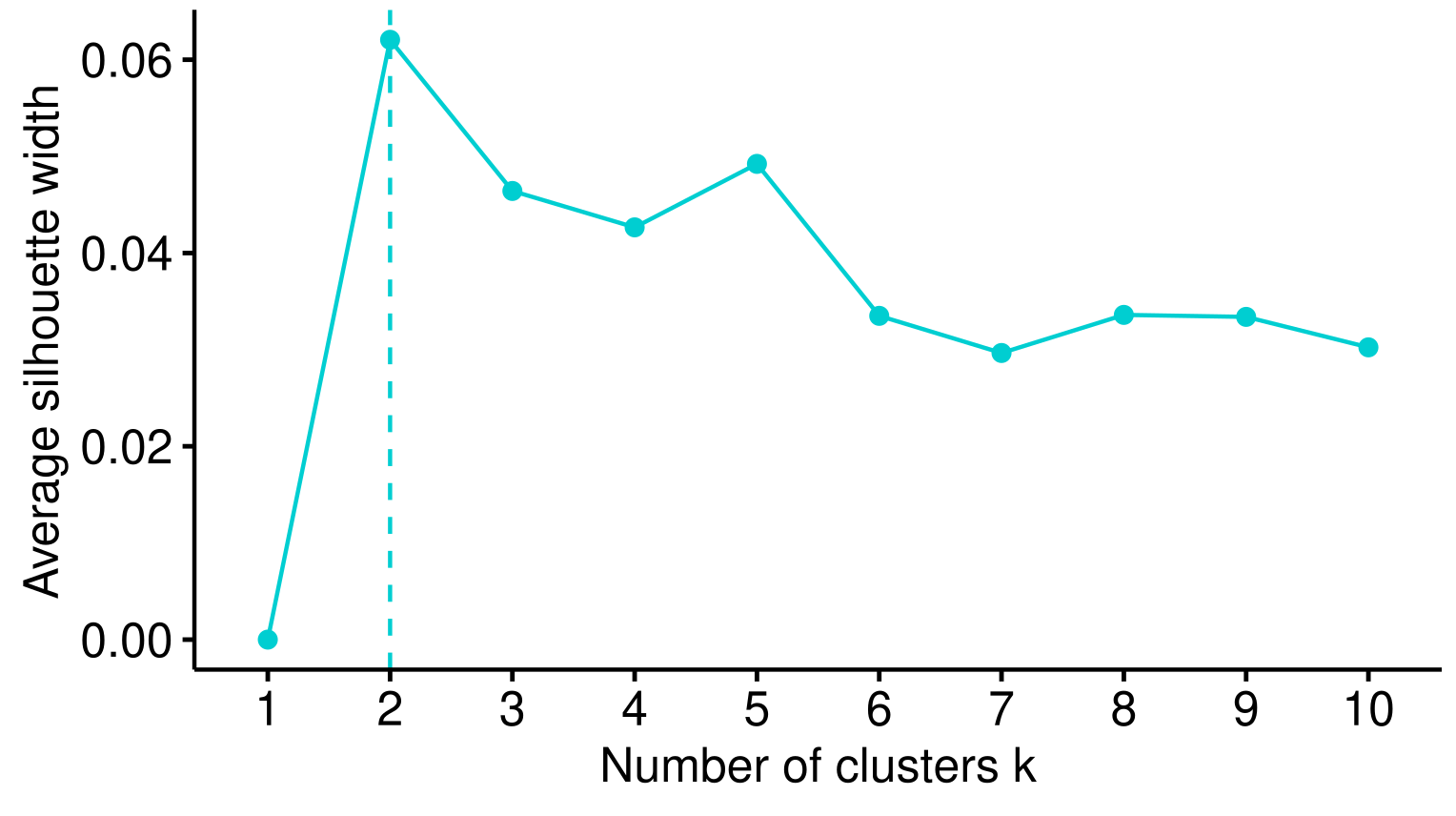

Supplement: S2 Fig — Optimal number of clusters (dashed line) for obtained family units from microbiome data of sows and their piglets at different production time points. (TIF) [file pone.0256112.s010.tif]

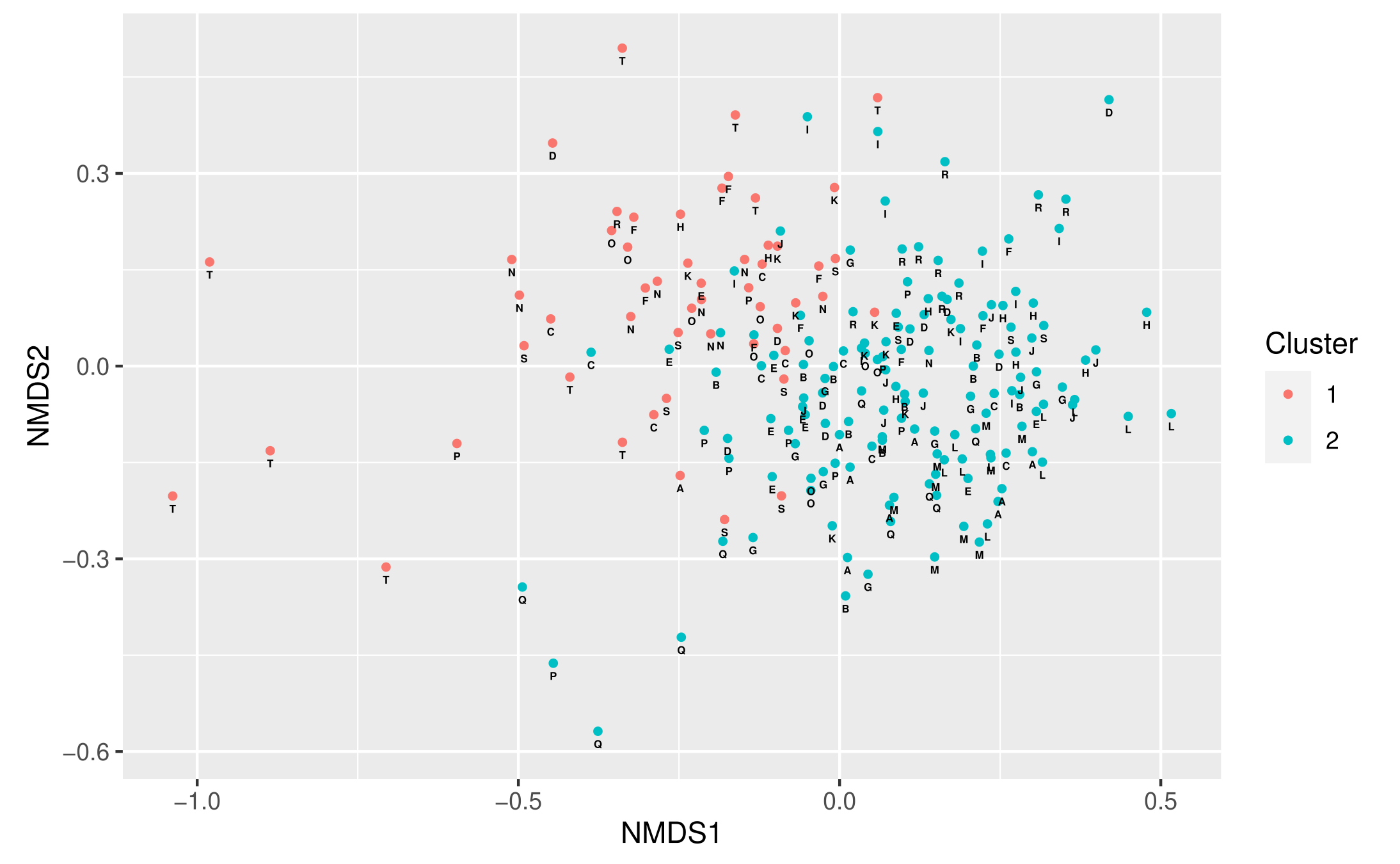

Supplement: S3 Fig — Each data point visualizes connected data of the sampling time points AP and PP for each sow in each farm. A–T = Individual farms. Different colors visualize cluster formation according to the optimal cluster formation method (S4 Fig). (TIF) [file pone.0256112.s011.tif]

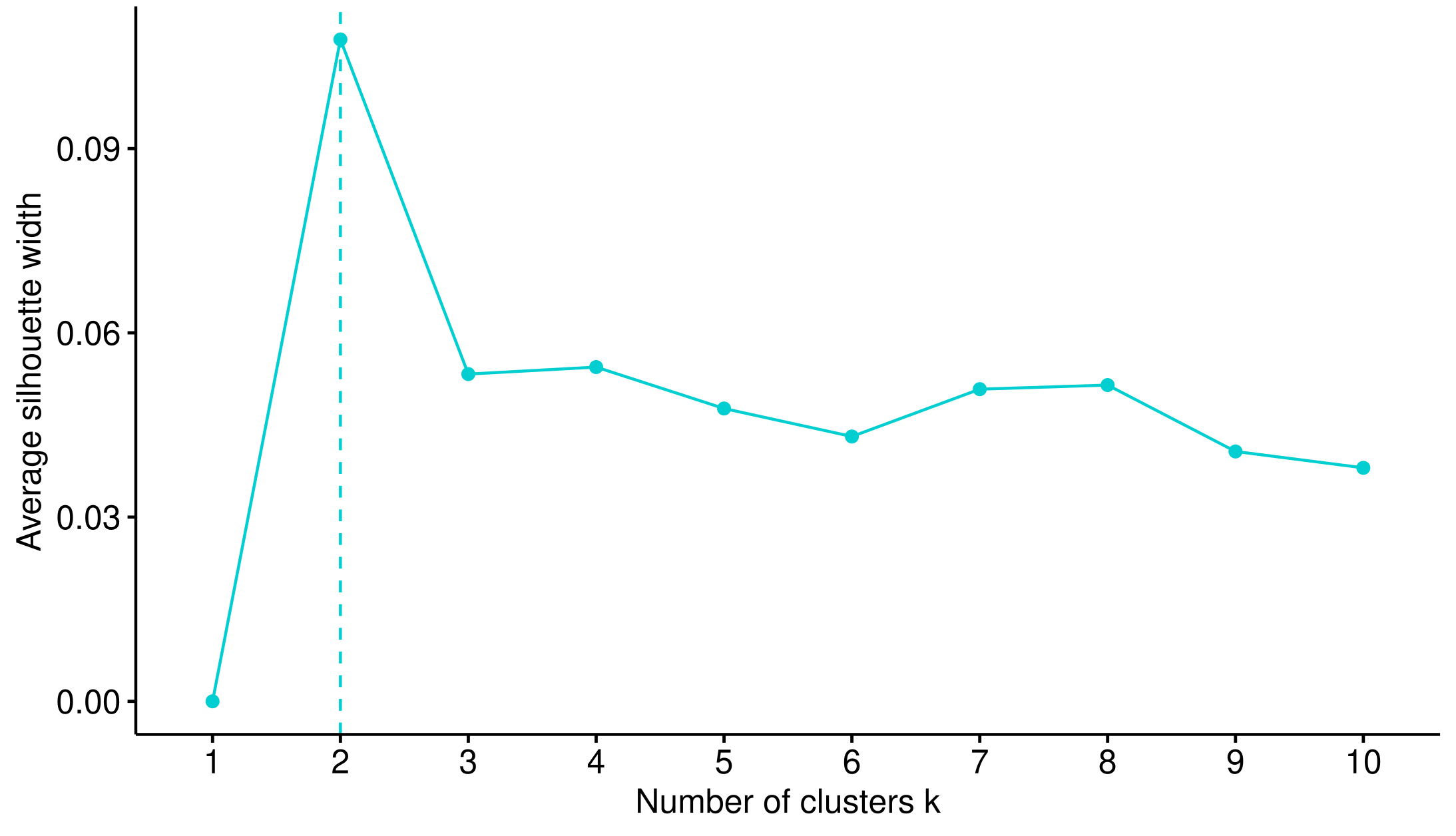

Supplement: S4 Fig — Optimal number of clusters (dashed line) for obtained sow units (AP and PP) from microbiome data of sows each farm. (TIF) [file pone.0256112.s012.tif]

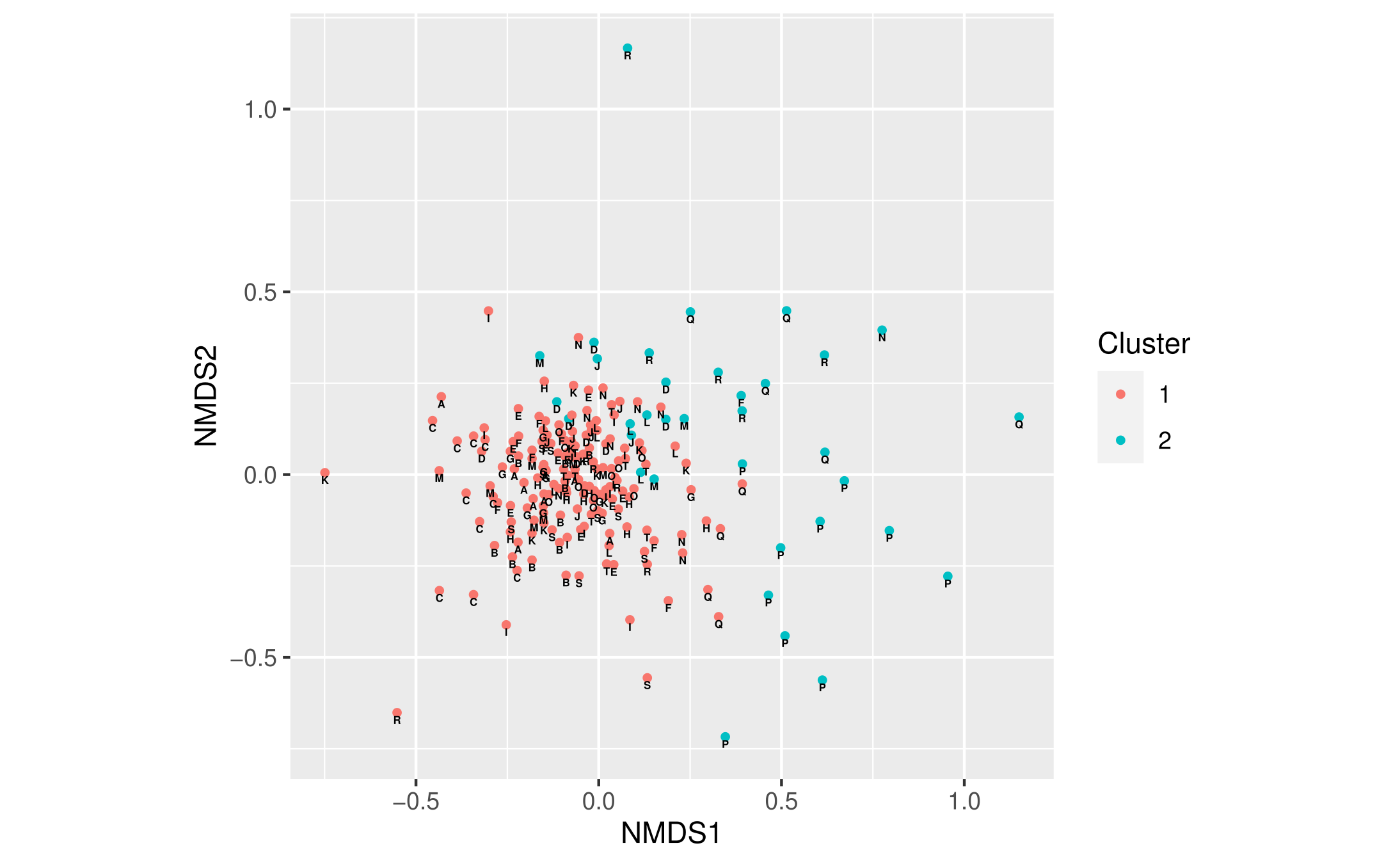

Supplement: S5 Fig — Each data point visualizes connected data of the sampling time points SP and PW for each piglet in each farm. A–T = Individual farms. Different colors visualize cluster formation according to the optimal cluster formation method (S6 Fig). (TIF) [file pone.0256112.s013.tif]

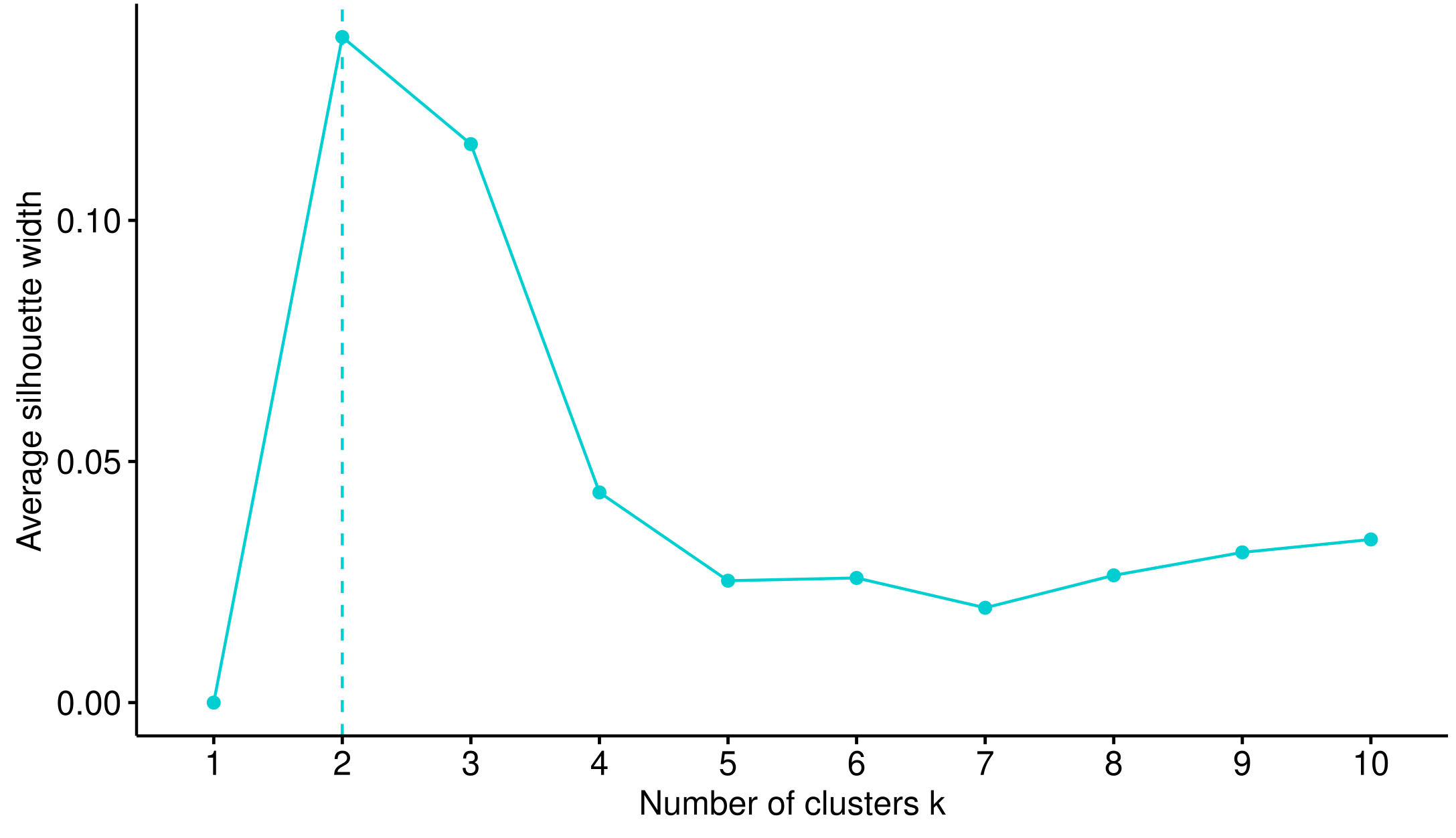

Supplement: S6 Fig — Optimal number of clusters (dashed line) for obtained piglet units (SP and PW) from microbiome data of piglets each farm. (TIF) [file pone.0256112.s014.tif]
